# Pharmacokinetics, safety, and tolerability of an oxfendazole tablet formulation: a phase 1, randomized, placebo-controlled trial in healthy African volunteers

**DOI:** 10.1128/aac.01315-25

**Published:** 2026-02-18

**Authors:** Said Jongo, Eveline Ackermann, Gloria Nyaulingo, Hussein Mbaraka, Elisabeth Reus, Silvia Cicconi, Meera Saxena, Kamaka Kassim, Anneth Tumbo, Mohammed Ally Rashid, Stephen Robinson, Mathieu Louis, Vijay Satam, Fabiana Barreira Da Silva Rocha, Ivan Scandale, Frauke Assmus, Jennifer Keiser, Sabine Specht

**Affiliations:** 1 Ifakara Health Institute 298427, Ifakara, Tanzania; 2 Swiss Tropical and Public Health Institute 30247, Allschwil, Switzerland; 3 University of Basel 27209, Basel, Switzerland; 4 Drugs for Neglected Diseases initiative 58076, Geneva, Switzerland; 5 Mahidol Oxford Tropical Medicine Research Unit, Faculty of Tropical Medicine, Mahidol University 115374, Bangkok, Thailand; 6 Centre for Tropical Medicine and Global Health, Nuffield Department of Medicine, University of Oxford 105596, Oxford, United Kingdom; Providence Portland Medical Center, Portland, Oregon, USA

**Keywords:** oxfendazole, onchocerciasis, tablet, healthy African volunteers, pharmacokinetics

## Abstract

Oxfendazole, a registered veterinary drug, has demonstrated broad-spectrum activity against multiple nematode species. First-in-human studies in healthy, predominantly Caucasian adults receiving a liquid formulation of oxfendazole demonstrated a favorable pharmacokinetic and safety profile, supporting its potential as a drug candidate for treating human helminth infections. To further advance its clinical development, we conducted a phase I bioavailability study using a field-applicable, immediate-release oxfendazole tablet. This trial investigated the pharmacokinetics, safety, and tolerability of the tablet formulation in healthy African adults residing in a filariasis-endemic country. Oxfendazole was administered as a single dose (100 or 400 mg) or as multiple doses (400 mg for 5 consecutive days) to 30 participants, randomized 8:2 to oxfendazole or placebo per cohort. Plasma concentrations of oxfendazole were measured using a validated high-performance liquid chromatography tandem mass spectrometry method, and pharmacokinetics were assessed using non-compartmental analysis. Peak plasma concentrations were reached after ~2.5–3 h. The median elimination half-life ranged from 11.6 to 13.9 h and was consistent across cohorts. Non-linear pharmacokinetics was observed, with exposure (AUC_∞_, *C*_max_) increasing less than dose-proportionally and showing high variability. Oxfendazole was well tolerated, with no adverse events reported and no clinically significant abnormalities in laboratory tests, vital signs, physical examinations, or electrocardiograms. These findings support the further development of oxfendazole in early proof-of-concept studies. Formulation optimization is suggested to reduce variability in exposure and improve the reliability of exposure-response assessments in future clinical trials.

The study is registered with ClinicalTrials.gov as NCT04920292.

## INTRODUCTION

Only a limited number of drugs are available for the treatment of neglected tropical diseases, particularly helminth infections caused by soil-transmitted helminths (STH) and filarial worms. These include ascariasis, trichuriasis, hookworm infections, lymphatic filariasis, and onchocerciasis—infectious diseases that cause significant morbidity and disproportionately affect the world’s poorest populations. Global efforts to eliminate human nematode infections, or provide individual patient care, are hampered by the absence of effective vaccines and macrofilaricidal drugs capable of killing adult worms with a short treatment regimen. Hence, there is an urgent need for new broad-spectrum anthelmintics that are safe, effective, and accessible in resource-limited settings.

Repurposing veterinary anthelmintics has emerged as a promising strategy to accelerate the development of new treatments. Oxfendazole is a benzimidazole anthelmintic with broad-spectrum activity against multiple parasites. It is widely used in veterinary medicine as oral and topical formulations for the treatment of gastrointestinal roundworms, lungworms, and tapeworms in cattle, sheep, horses, and dogs (1). More specifically, oxfendazole has shown high efficacy against tissue-dwelling larval infections caused by *Taenia solium* (porcine cysticercosis), *Echinococcus granulosus*, and *Fasciola hepatica* in sheep (1). In experimental models of filarial infections, it was effective against *Litomosoides sigmodontis*, *Brugia* spp., and *Acanthocheilonema viteae* (2). It also demonstrated potent activity against *Heligmosomoides polygyrus*, a mouse nematode commonly used as a model for human hookworm infections (3). Oxfendazole has been approved for veterinary use in the United States, the European Union, and Morocco and is under consideration for registration in several African countries.

Like other benzimidazole drugs, oxfendazole disrupts parasite microtubules and is postulated to irreversibly block glucose uptake in parasites, depleting their energy stores and ultimately causing death. Notably, this mechanism appears specific to parasites and does not affect glucose metabolism in mammals.

Oxfendazole (fenbendazole sulfoxide) is metabolized through sulfur oxidation to oxfendazole sulfone and sulfur reduction to fenbendazole. The conversion to fenbendazole is reversible, as demonstrated in both *in vivo* and *in vitro* studies. Importantly, oxfendazole shows ~40% higher oral bioavailability than fenbendazole and therefore achieves higher systemic exposure (4, 5). Oxfendazole is further characterized by a long metabolic half-life in ruminants; its active metabolite fenbendazole also exhibits anthelmintic activity (1, 6).

Beyond its broad-spectrum anthelminthic potential, oxfendazole has the added advantage of lacking direct activity against microfilariae, thereby minimizing the risk of microfilariae-induced inflammatory responses in patients with loiasis and onchocerciasis. This is expected to prevent drug-induced, microfilarial-dependent serious adverse events (SAEs) frequently observed with diethylcarbamazine citrate or ivermectin treatment (7, 8). Given its distinct pharmacological profile, oxfendazole is a promising candidate for expanding the limited portfolio of antiparasitic drugs for treating multiple helminth infections in humans, including onchocerciasis, loiasis, mansonellosis, and the STHs.

Oxfendazole has already been tested in two phase I clinical trials using a veterinary liquid formulation: a first-in-human single ascending dose (SAD) (9) and a multiple ascending dose (MAD) study in healthy volunteers (10). Oxfendazole was found to be safe and well tolerated following oral administration at single doses up to 60 mg/kg and daily doses of up to 15 mg/kg for 5 days. Adverse events (AEs) were reported in 14% of participants in the SAD study and 31% in the MAD study, mostly mild and transient, with few moderate events. Reported AEs mainly involved nervous system and gastrointestinal symptoms, as well as metabolic disorders. Potentially efficacious plasma exposure following oral dosing was observed, confirming a favorable pharmacokinetic (PK) profile and oral bioavailability, while also revealing non-linear pharmacokinetics likely due to solubility-limited absorption.

To support future efficacy studies in endemic settings, a field-applicable, immediate-release tablet formulation of oxfendazole was developed. Here, we report the PK, safety, and tolerability of this new tablet formulation in healthy adults from sub-Saharan Africa (Tanzania) after single and multiple oral doses, evaluating its suitability for early proof-of-concept studies. Incorporating African populations early in drug development is critical to ensure that new therapeutics are appropriate for and applicable to the settings where their use is most relevant (11).

## MATERIALS AND METHODS

### Study design

This was a randomized, placebo-controlled, double-blind, single-center phase I bioavailability study, evaluating the PK, safety, and tolerability of an oxfendazole tablet formulation in healthy adults. The study was conducted at the Bagamoyo Clinical Trial Facility of the IHI in Tanzania between 21 April 2022 (first participant screened) and 14 November 2022 (last participant last visit).

The primary objective was to investigate the PK of oxfendazole and its metabolites, fenbendazole and oxfendazole sulfone, after single and multiple oral administrations of the tablet formulation. The secondary objective was to evaluate the safety and tolerability of oxfendazole.

Thirty participants were randomized 8:2; 3 cohorts of 10 participants were sequentially recruited to receive oxfendazole or placebo. In each cohort, the first two participants were hortrandomized using a sentinel approach, such that one participant received a dose of oxfendazole and the other a placebo. Participants in cohort one received a single dose of 100 mg of oxfendazole (corresponding to 1.7 mg/kg for a 60 kg participant). Participants in cohort 2 received a single dose of 400 mg of oxfendazole (corresponding to 6.7 mg/kg for a 60 kg participant). Participants in cohort 3 received multiple doses of 400 mg/day of oxfendazole over 5 days (corresponding to 6.7 mg/kg/day for a 60 kg participant). Oxfendazole was administered as a 100 mg immediate-release tablet or a visually matching placebo, both manufactured by Syngene (Bengaluru, India). Details on tablet composition and dissolution characteristics are provided in Information S1 . All participants fasted for at least 8 h before and for 2 h after the intake of the study drug.

The maximum dose of 400 mg was selected based on exposure levels from previous phase 1 studies (9, 10) and predicted efficacious exposure derived from preclinical studies in (7). This dose was considered adequate to achieve exposure levels relevant for potential therapeutic use.

To minimize risks, the study drug was administered sequentially within each cohort using a sentinel approach, as described above and detailed in Information S2: the first two participants received either oxfendazole or placebo. Following a minimum safety surveillance period of 48 h for the single dose cohorts and 72 h after the last dose for the multiple dose cohort, safety was evaluated by the investigator based on clinical and laboratory data. The remaining eight participants were subsequently enrolled. Progression to the next cohort was permitted only after the Safety Review Committee confirmed that the preceding dose was safe and well tolerated.

Each participant was followed up for up to 14 days following the initial dose. Participants in cohort 1 and cohort 2 were observed on-site for 2.5-day post-dose, while participants in cohort 3 were observed for 3.5 days after taking the last dose. A detailed schedule of events is provided in Information S3.

The study was conducted in a double-blind manner: participants, site staff, sponsor staff, the central electrocardiogram (ECG) reader, the PK expert (responsible for the PK calculations), the study monitor(s), and the study statistician were blinded to the treatment allocation. Only the independent statistician, site pharmacist, and bioanalytical lab personnel analyzing drug levels were unblinded. To maintain blinding, an independent monitor handled drug monitoring and accountability.

Recruitment was performed with locally applicable and Independent Ethics Committee-approved methods of advertising (e.g., fliers and announcements) and sensitization meetings with community members, ward leaders, community health workers, and key opinion leaders in the community. Discussions on study procedures, risks, and potential benefits were primarily conducted in Swahili (local language), and all explanations were given in a culturally appropriate manner.

### Study participants

Healthy adult participants residing in the Bagamoyo area of Tanzania—a region endemic for filariasis and STHs—were enrolled in the study. Participants were eligible if they were male or female adults (non-pregnant and non-breastfeeding), 18 to 45 years of age, with a body mass index (BMI) between 18 and 29.9 kg/ m^2^), and deemed healthy based on the absence of abnormal physical findings, ECG results, and clinically significant laboratory abnormalities. Women of childbearing potential were required to use a highly effective form of contraception in combination with a barrier method (e.g., condom). Men were required to use condoms until 90 days after last dosage. Blood, stool, and urine samples were analyzed for exclusion of helminth infestations (e.g., onchocerciasis, [neuro-]cysticercosis, and echinococcosis), malaria, and other chronic conditions such as HIV and hepatitis. Further key exclusion criteria were presence or history of drug or alcohol abuse, regular smoking (more than five cigarettes per day), recent use of medication, and use of dietary supplements or herbal remedies known to interfere with P-glycoprotein and/or the CYP3A4 metabolic pathway. Finally, volunteers were excluded if they had a history of drug allergies or showed hypersensitivity to benzimidazoles or the tablet excipients.

### Study procedures

#### Safety assessments

Safety and tolerability were assessed through monitoring of AEs, physical examinations, vital signs, ECGs, and clinical laboratory tests. All AEs and SAEs—except for laboratory abnormalities and vital signs—were graded using a 1–5 scale (mild, moderate, severe, life-threatening, and death). The AE collection period started after administration of the first dose and continued until the final follow-up visit on Day 14. Participants were monitored for AEs daily during the ward period and were enquired about any ongoing or newly observed AEs since the previous assessment. An AE was defined as any untoward medical occurrence in a participant administered the study drug or placebo, irrespective of causal relationship. Laboratory or vital sign abnormalities were only recorded as AEs if assessed by the investigator as clinically significant.

Laboratory assessments were performed according to the schedule of assessments and standard operating procedures of the local laboratory. These included (i) biochemistry: creatinine, alanine aminotransferase (ALT), and aspartate aminotransferase (AST), and total bilirubin, sodium, potassium, chloride, bicarbonate, blood urea nitrogen, and glucose (only at screening); (ii) hematology and coagulation: hemoglobin, white blood cell (WBC) count (differentiation of eosinophils and neutrophils), platelets, prothrombin time, and activated partial thromboplastin time (aPTT); (iii) urinalysis performed by dipstick: proteinuria and glycosuria, (iv) pregnancy testings, and (v) serology. Laboratory abnormalities and vital sign deviations were graded according to the site’s grading scale, adapted from the US Food and Drug Administration guidance for industry.

Standard 12-lead ECGs were recorded at screening and on Day 1 (pre-dose and 1, 2, 3 h post-dose, cohorts 1, 2, and 3) and on Day 5 (pre-dose and 1, 2, 3 h post-dose, cohort 3). At each time point, three repeat ECGs (at least 1 min apart) were performed. ECG parameters included heart rate (HR), PR interval, QRS duration, QT interval, cardiac rhythm, and T wave morphology. QT intervals were corrected using Fridericia’s (QTcF) formula. Screening ECGs were reviewed by study clinicians, with cardiologist input as needed; all post-baseline ECGs were reviewed centrally by a cardiologist. Any abnormalities confirmed by repeated measurements were assessed for clinical significance.

#### PK sample collection

Blood samples for PK analysis of oxfendazole and its metabolites were collected at the following time points: In cohorts 1 and 2, samples were drawn pre-dose and at 1, 2, 3, 4, 6, 8, 10, 12, 24, and 48 h post-dose. In cohort 3, samples were collected pre-dose and at 1, 2, 3, 4, 6, 8, 10, and 12 h after dosing on Day 1; pre-dose and 2 h after dosing on Days 2 and 3; pre-dose on Day 4; and pre-dose and at 1, 2, 3, 4, 8, 12, 24, 48, and 72 h following the final dose on Day 5. Plasma samples were shipped on dry ice to Swiss BioQuant AG (Reinach, Switzerland) for drug quantification.

### Bioanalysis

Plasma concentrations of oxfendazole, fenbendazole, and oxfendazole sulfone were quantified using a validated high-performance liquid chromatography tandem mass spectrometry method. Details of the analytical procedure, including instrumentation, gradient conditions, and assay performance, are provided in Information S4.

### PK analysis

The PK analysis population included all participants who received a dose of the study drug and had at least one quantifiable plasma concentration of oxfendazole and/or its metabolites above the lower limit of quantification (LLOQ), without relevant protocol deviations likely to impact PK interpretation. The PK parameters of oxfendazole and its metabolites in plasma were described using non-compartmental analysis (NCA) in Phoenix WinNonlin (Version 8.3). Plasma concentrations below the LLOQ (BLQ) were set to zero. BLQ values between two measurable concentrations were set to missing and excluded from analysis without imputation.

The following PK parameters were estimated from individual plasma concentration-time profiles using the actual sampling times on Days 1 and 5 (cohort 3 only): area under the plasma concentration curve from time zero to the last quantifiable concentration at time *t* (AUC_0-*t*_), AUC from time zero to infinity with extrapolation of the terminal phase (AUC_0-∞_), AUC during a dosing interval (AUC_tau_), maximum plasma concentration (*C*_max_), time to reach *C*_max_ (*T*_max_), apparent elimination half-life (*t*_1/2_), and accumulation ratio (Racc, calculated as AUC_tau, Day 5_/AUC_tau, Day 1_). AUC_tau_ refers to the area under the curve over a 24-h interval.

*C*_max_ and *T*_max_ were obtained directly from the concentration-time data. The terminal phase rate constant (λz) was estimated by linear regression of the log-transformed concentration-time data from the terminal phase, and *t*_1/2_ was derived as ln (2)/λz. AUCs were calculated using the linear up/log down trapezoidal method.

### Statistical analysis

All statistical analyses were descriptive, and no formal comparisons were made between dose groups or cohorts. Data for participants receiving oxfendazole were presented by cohort (cohorts 1, 2, and 3), while placebo data were pooled across cohorts.

Demographic and baseline characteristics were summarized as arithmetic means (with standard deviation, SD) or medians (minimum to maximum range) for continuous variables and as counts (percentages) for categorical variables. Baseline was defined as the latest assessment available prior to study drug administration. No covariate adjustments were made due to the exploratory nature of the study.

PK parameters were summarized as median (range) for *C*_max_, AUC, *t*_1/2_, *T*_max_, and Racc. Descriptive statistics for PK parameters, based on actual blood sampling time points, were calculated with Phoenix WinNonlin. PK plots showing individual and median concentration-time profiles (based on actual sampling times), as well as boxplots of PK parameters, were generated in R (v4.2.2). All summaries were based on non-missing values, with the number and percentage of missing values reported, if applicable. PK parameters were not summarized when fewer than three non-missing values were available (*N* < 3).

Statistical analyses of demographics and safety data were performed using R (Version 4.2.2), and ECG analyses were conducted using SAS (Version 9.4).

## RESULTS

### Baseline characteristics of the study population

**Fig 1:**
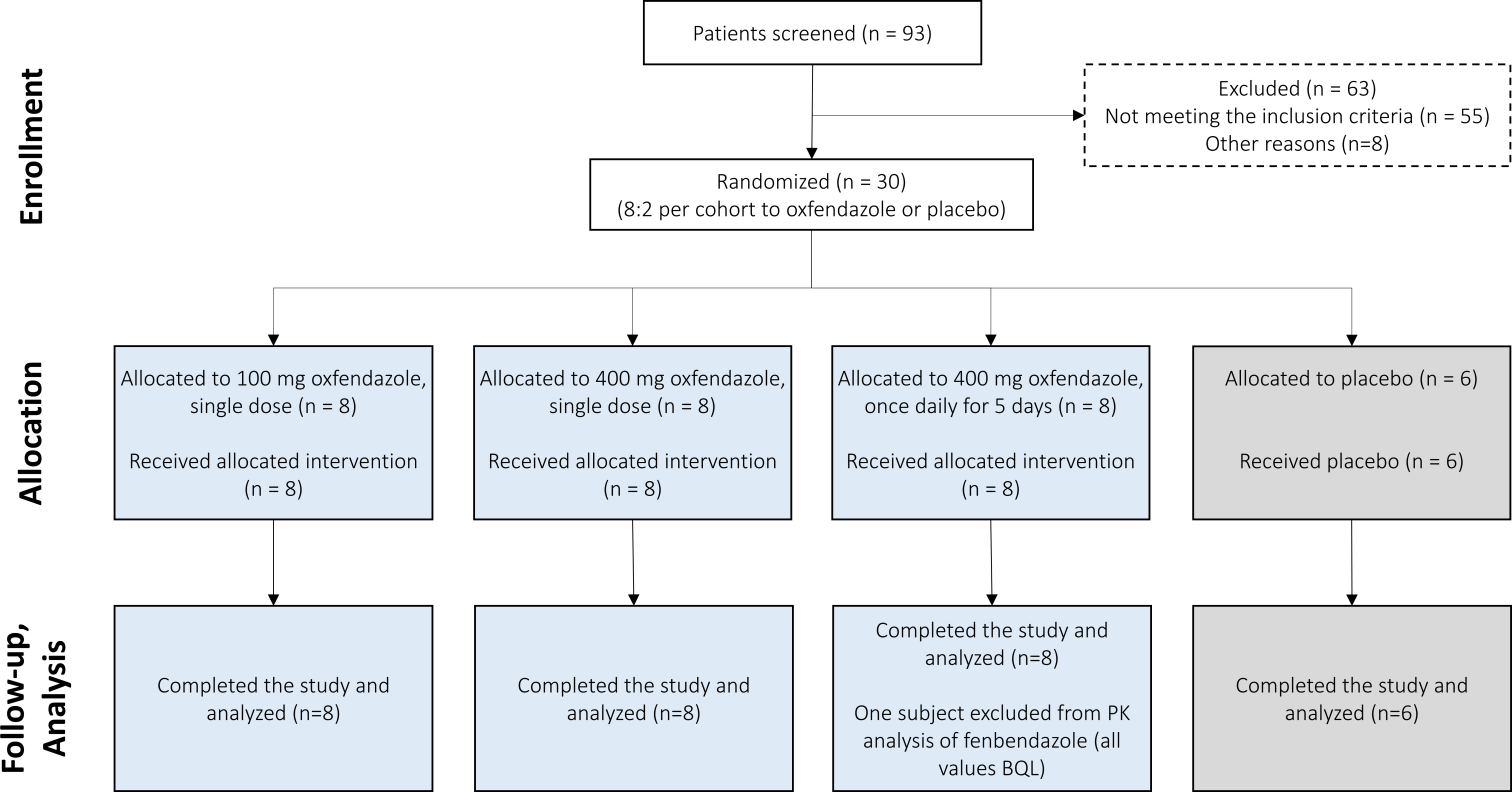
Flow diagram of participant enrollment, allocation, and analysis. A total of 93 participants were screened and provided informed consent. A total of 30 participants were randomized 8:2 ratio to oxfendazole or placebo across three cohorts. All participants completed the study. One participant in the 400 mg single-dose cohort was excluded from the PK analysis of fenbendazole due to all plasma concentrations being BLQ.

In this phase I study, a total of 93 individuals were screened, of whom 30 healthy adults were enrolled and randomized. All randomized participants completed the study. Within each of the 3 cohorts, 8 participants were randomized to receive oxfendazole and 2 to receive placebo, resulting in 24 participants receiving oxfendazole and 6 receiving placebo overall. The treatment arms included two single-dose cohorts (100 and 400 mg oxfendazole) and one multiple-dose cohort (400 mg oxfendazole once daily for 5 days). Participant enrollment, allocation, and inclusion in the final analysis are summarized in Fig. 1.

Demographic and baseline laboratory characteristics of the study participants are provided in Table 1. The study population included 18 males (60.0%) and 12 females (40.0%), all healthy adults of Black African ethnicity residing in Tanzania. The mean (SD) age was 28.0 (5.4) years, and the mean (SD) BMI was 22.2 (3.3) kg/m^2^. Most baseline laboratory values—including hemoglobin, white blood cell counts, and neutrophil and eosinophil levels—were within established reference ranges. Minor deviations observed in individual participants were not considered clinically significant. No apparent differences between treatment groups were observed in any of these parameters.

**TABLE 1:** Demographic and baseline laboratory characteristics of the participants included in the HELP-OFZ study^*c*^

Characteristic	Cohort 1 100 mg OXF (*N* = 8)	Cohort 2 400 mg OXF (*N* = 8)	Cohort 3 400 mg OXF, 5 days (*N* = 8)	Placebo pooled (*N* = 6)	Reference range
Sex, *n* (%)					
Female	3 (37.5)	3 (37.5)	4 (50.0)	2 (33.3)	
Male	5 (62.5)	5 (62.5)	4 (50.0)	4 (66.7)	
Ethnicity, *n* (%)					
Black African	8 (100%)	8 (100%)	8 (100%)	6 (100%)	
Age [years]^*a*^	30.2 ± 4.0 (24–38)	26.4 ± 7.0 (19–41)	26.2 ± 5.0 (19–33)	29.3 ± 5.0 (24–38)	
Weight [kg]^*a*^	56.9 ± 3.9 (49–61)	59.5 ± 10.5 (42–75)	58.9 ± 7.3 (49–74)	57.7 ± 15.8 (48–89)	
BMI [kg/m^2^]^*a*^	20.4 ± 1.5 (18.3–22.3)	23.5 ± 3.7 (18.4–28.9)	22.5 ± 3.6 (18.9–28.9)	22.4 ± 3.5 (20.2–29.4)	
ALT [U/L]^*b*^	12.2 (8.2–21.8)	20.6 (7.8–28.0)	10.9 (8.8–53.6)	14.2 (6.9–57.5)	3.5–46.8
AST [U/L]^*b*^	17.9 (12.6–22.5)	23.6 (13.3–27.7)	16.3 (10.7–36.6)	19.1 (12.7–32.1)	12.3–74.7
Bilirubin [μmol/L]^*b*^	11.1 (5.1–18.2)	8.1 (3.6–10.6)	7.2 (4.8–28.4)	8.5 (4.8–17.8)	3.1–31.1
Creatinine [μmol/L]^*b*^	57.0 (49.0–70.0)	64.0 (38.0–93.0)	63.0 (40.0–74.0)	69.5 (42.0–94.0)	49–95.3
Hemoglobin [g/dL]^*b*^	13.6 (11.0–16.3)	15.0 (12.0–15.9)	13.2 (11.6–15.2)	13.7 (12.8–14.1)	Female: 9.6–14.1 Male: 12.6–17.3
WBC count [10^3^/μL]^*b*^	5.0 (4.1–5.9)	4.8 (3.2–6.4)	5.0 (3.0–9.0)	5.3 (3.8–5.9)	3.48–9.11
Neutrophils [10^3^/μL]^*b*^	2.2 (1.5–3.0)	2.0 (1.3–3.1)	2.2 (0.8–7.0)	2.3 (1.4–3.9)	1.18–5.46
Eosinophils [10^3^/μL]^*b*^	0.15 (0.07–0.32)	0.09 (0.03–0.34)	0.08 (0.06–0.52)	0.11 (0.04–0.20)	0–0.78
Platelets [10^3^/μL]^*b*^	220 (110–275)	250 (107–379)	227 (199–319)	268 (207–285)	107–396.2
Prothrombin (sec)	10.3 (9.7–11.9)	10.5 (10.0–11.0)	10.2 (9.5–11.6)	10.6 (9.5–11.8)	9.9–11.8
aPTT (sec)	36.0 (29.5–39.8)	33.3 (29.3–37.9)	33.8 (30.6–37.8)	33.6 (29.7–42.0)	26.1–36.3

^*a*^ Demographics at consent presented as mean ± SD (minimum–maximum).

^*b*^ Laboratory parameters presented as median (minimum–maximum).

^*c*^ OXF, oxfendazole; BMI, body mass index; ALT, alanine amino-transferase; AST, aspartate aminotransferase.

### PK analysis

**Fig 2:**
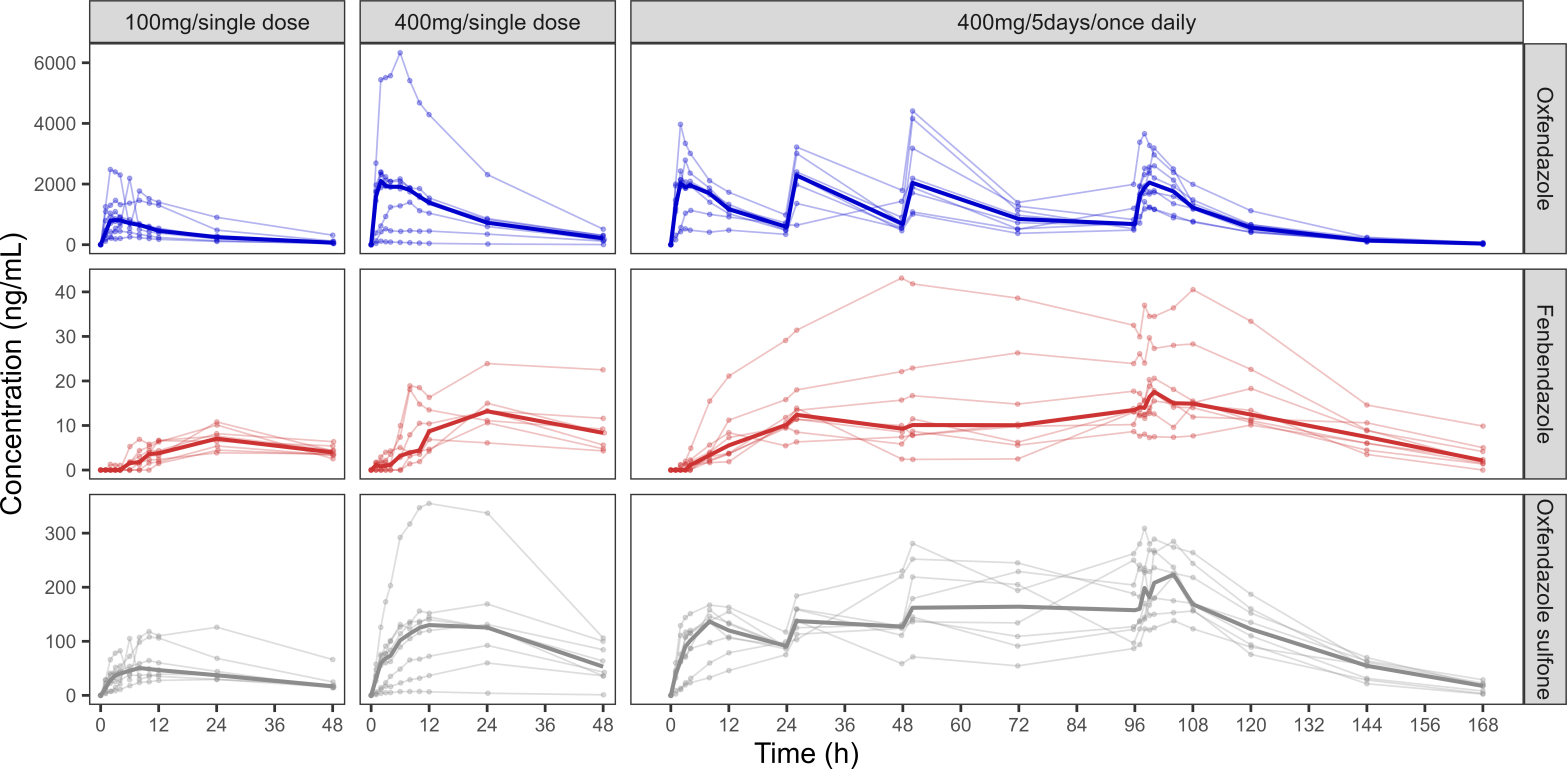
Individual plasma concentration-time profiles of oxfendazole and its metabolites, fenbendazole and oxfendazole sulfone, in healthy adult participants (*n* = 8 per cohort) following administration of oxfendazole as a single 100 mg dose (cohort 1), single 400 mg dose (cohort 2), or 400 mg once daily for 5 days (cohort 3). Thin lines represent individual participant profiles; bold lines represent the median concentration at each time point. For fenbendazole, all PK samples from one participant in cohort 2 were below the limit of quantification and are not shown (*n* = 7 for fenbendazole in cohort 2).

All 24 participants who received oxfendazole were included in the PK analysis. All plasma concentrations of oxfendazole and oxfendazole sulfone were above the LLOQ and included in the descriptive statistics. For fenbendazole, 83 of 328 plasma concentrations (25.3%) were BLQ, primarily during the formation phase. In one participant in the 400 mg/single dose cohort, all fenbendazole plasma levels were BLQ; this individual was excluded from the PK summary statistics for fenbendazole, and no PK profile is shown. Individual plasma concentration-time profiles of oxfendazole, fenbendazole, and oxfendazole sulfone across cohorts, along with median concentrations, are shown in Fig. 2. Corresponding PK profiles on a semi-logarithmic scale are provided in Information S5. For completeness, arithmetic mean concentrations with SDs at nominal time points are also shown in Information S6. A summary of descriptive PK parameters is presented in Table 2.

**TABLE 2:** PK parameters for oxfendazole and its metabolites fenbendazole and oxfendazole sulfone^*a*^

PK parameter	Cohort 1 (100 mg, single dose)	Cohort 2 (400 mg, single dose)	Cohort 3 (400 mg, 5 days once daily)	
			Day1	Day5
*Oxfendazole*				
*C*_max_ (ng/mL)	1,070 (256–2,480)	2,130 (117–6,330)	2,140 (523–3,970)	2,150 (1,240–3,660)
*T*_max_ (h)	3.06 (2.00–6.05)	2.53 (2.00–8.00)	2.53 (2.00–4.13)	3.02 (2.00–4.03)
AUC_0-*t*_ (ng × h/mL)	15,700 (5,970–45,300)	44,100 (1,460–126,000)		
AUC_0-∞_ (ng × h/mL)	16,900 (7,320–52,700)	47,000 (1,520–135,000)		
AUC_tau_ (ng × h/mL)			28,700 (9,850–44,200)	31,600 (18,500–50,300)
*t*_½_ (h)	12.9 (9.85–18.7)	13.9 (10.1–21.3)	NE	11.6 (8.97–15.4)
*R*acc	–^*b*^	–	–	1.15 (0.64–3.02)
*Fenbendazole*				
*C*_max_ (ng/mL)	7.09 (3.86–10.8)	13.3 (6.86–23.9)	10.2 (8.38–29.1)	18.8 (10.1–40.5)
*T*_max_ (h)	24.1 (12.0–24.3)	24.0 (8.00–24.1)	23.8 (12.0–23.8)	4.02 (2.97–24.0)
AUC_0-*t*_ (ng × h/mL)	250 (131–273)	410 (221–915)		
AUC_0-∞_ (ng × h/mL)	NE	NE		
AUC_tau_ (ng × h/mL)			119 (87.7–412)	344 (197–873)
*t*_½_ (h)	NE	NE	NE	NE
*R*acc	–	–	–	3.27 (1.46–3.74)
*Oxfendazole sulfone*				
*C*_max_ (ng/mL)	58.1 (29.4–126)	136 (7.19–355)	141 (75.1–167)	241 (138–309)
*T*_max_ (h)	10.0 (6.00–24.1)	18.0 (10.0–24.1)	10.0 (7.98–23.8)	3.01 (0.00–8.05)
AUC_tau_ (ng × h/mL)	1,560 (1,120–4,550)	4,810 (180–11,900)		
AUC_∞_ (ng × h/mL)	2,160 (1,880–3,530)	NE		
AUC_tau_ (ng × h/mL)			2,490 (1,030–3,340)	4,050 (2,750–6,030)
*t*_½_ (h)	20.8 (17.0–28.4)	NE	NE	15.9 (8.97–24.1)
*R*acc	–	–	–	1.76 (1.03–4.80)

^*a*^ Data are median (minimum–maximum). *C*_max_, maximum plasma concentration; *T*_max_, time to *C*_max_. AUC_0-*t*_, area under the plasma concentration curve from time zero to the last quantifiable concentration at time t (48 h, for cohorts 1 and 2). AUC_0-∞_, AUC from time zero to infinity with extrapolation of the terminal phase. AUC_tau_, area under the plasma concentration curve during a dose interval (24 h, for cohort 3). *t*_1/2_, elimination half-life; Racc: accumulation ratio. NE, not estimated or insufficient data because *N* < 3. N, 8 participants for all parameters except for fenbendazole in the 400 mg single-dose group, where *N* = 7. The number of participants differed for the parameters AUC_0-∞_ and *t*_1/2_ for oxfendazole sulfone (*N* = 5).

^*b*^ –, not applicable.

### Single-dose pharmacokinetics

After single-dose administration, oxfendazole exhibited slow absorption, with median *T*_max_ values of 3.06 h in the 100 mg cohort and 2.53 h in the 400 mg cohort (Table 2). Following the attainment of *C*_max_, plasma concentrations decreased slowly in a mono-phasic manner. The median terminal *t*_1/2_ of oxfendazole ranged from 12.9 to 13.9 h and appeared to be independent of dose. Within the small dose range investigated, systemic exposure increased less than proportionally with oxfendazole dose: a fourfold increase in dose resulted in only a twofold increase in *C*_max_ and a 2.8-fold increase in AUC_0-∞_.

**Fig 3:**
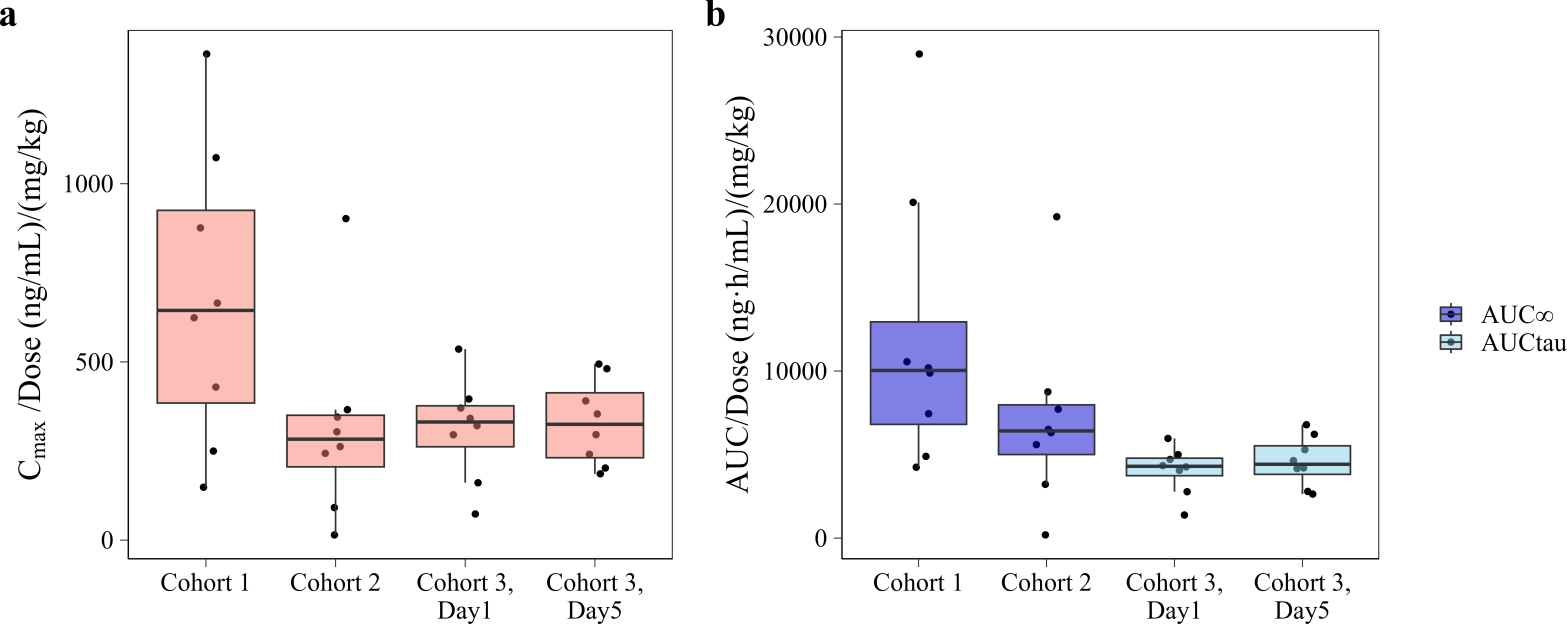
Dose-normalized exposure of oxfendazole across dosing cohorts. (**a**) Dose-normalized peak plasma concentration (*C*_max_/dose) and (**b**) dose-normalized area under the plasma concentration-time curve (AUC/dose) for each cohort. Cohort 1: 100 mg oxfendazole single dose; cohort 2: 400 mg single dose; cohort 3: 400 mg once daily for 5 days. Dose normalization was based on dose per kg body weight. Boxes represent the interquartile range (IQR), the bold line indicates the median, and whiskers extend to 1.5× IQR. Individual participant values are shown as dots. AUC values are stratified by calculation method: AUC_∞_ (from time zero to infinity) for single-dose cohorts and AUC_tau_ (during a 24-h dosing interval) for the multiple-dose cohort.

This is further illustrated in Fig. 3, which shows dose-normalized exposure (*C*_max_/dose and AUC/dose) across all cohorts. If pharmacokinetics were dose-proportional, these values would be comparable across cohorts. However, lower dose-normalized exposures were observed in the higher dose cohorts, reflecting less than dose-proportional increases in exposure.

The PK of oxfendazole was highly variable, particularly in the 400 mg cohort, where one participant exhibited very low (*C*_max_ = 117 ng/mL, AUC_0-∞_ = 1,520 ng × h/mL), and another very high exposure (*C*_max_ = 6,330 ng/mL, AUC_0-∞_ = 135,000 ng × h/mL). Notably, inter-individual variability remained substantial even after exclusion of these extremes.

Plasma concentrations of fenbendazole and oxfendazole sulfone were substantially lower than those of oxfendazole (Fig. 2, middle and lower panels). Of the two metabolites, exposure was higher for oxfendazole sulfone. The plasma concentration-time profiles indicated slow formation, with a median *T*_max_ of approximately 24 h for fenbendazole and 10–18 h for oxfendazole sulfone. Due to limited blood sampling, *t*_1/2_ for the metabolites could not be estimated for all participants. Similar to oxfendazole, metabolite exposure (*C*_max_, AUC) increased with dose but appeared less than proportional. Key PK parameters for oxfendazole and the metabolites, both in absolute terms and normalized to dose, are shown inInformation S7 . Inter-individual variability was high for both metabolites. The apparently lower variability of fenbendazole is attributable to the exclusion of one participant with all concentrations BLQ.

### Multiple-dose pharmacokinetics

After 3 days of dosing with 400 mg oxfendazole, steady state was reached for oxfendazole (Fig. 2, upper right panel). In contrast, trough concentrations of fenbendazole and oxfendazole sulfone continued to increase beyond Day 3, and attainment of steady state after 5 days could not be confirmed. After 5 days of dosing, the median *T*_max_ of oxfendazole was 3.02 h, and *t*_1/2_ was 11.5 h, similar to values observed after single-dose administration (Table 2). Exposure of fenbendazole and oxfendazole sulfone remained much lower than that of oxfendazole.

**Fig 4:**
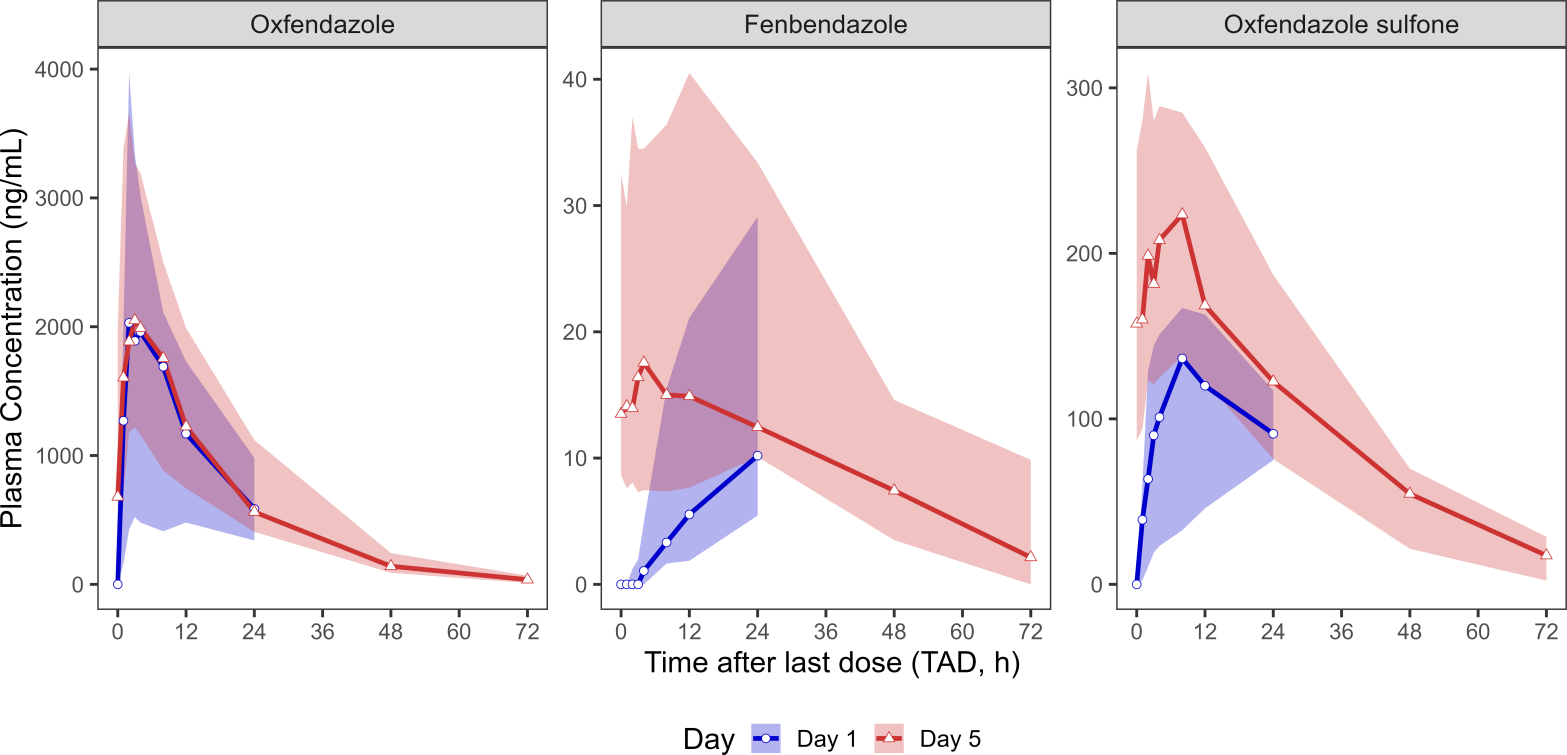
Median plasma concentration-time profiles of oxfendazole and its metabolites, fenbendazole and oxfendazole sulfone, in participants (*n* = 8) receiving 400 mg oxfendazole once daily for 5 days (Cohort 3). Data are shown for Day 1 (blue) and Day 5 (red) based on TAD. Lines represent median plasma concentrations at each nominal TAD; shaded areas indicate the observed range (minimum to maximum).

Figure 4 shows plasma concentrations over time after the last dose (TAD) on Day 1 versus Day 5. These profiles illustrate minor accumulation of oxfendazole (median Racc = 1.14), which likely contributed to the small differences observed in dose-normalized *C*_max_ and AUC_tau_ between Day 1 and Day 5 (Fig. 3). In contrast to oxfendazole, accumulation of the metabolites was more pronounced, with median Racc values of 1.76 for oxfendazole sulfone and 3.27 for fenbendazole. However, oxfendazole remained the main moiety in plasma.

PK variability of oxfendazole appeared somewhat lower after multiple dosing compared to single-dose administration, although direct comparison is limited by the small sample size (*N* = 8).

### Comparison with suspension (historical data in Caucasian volunteers)

**Fig 5:**
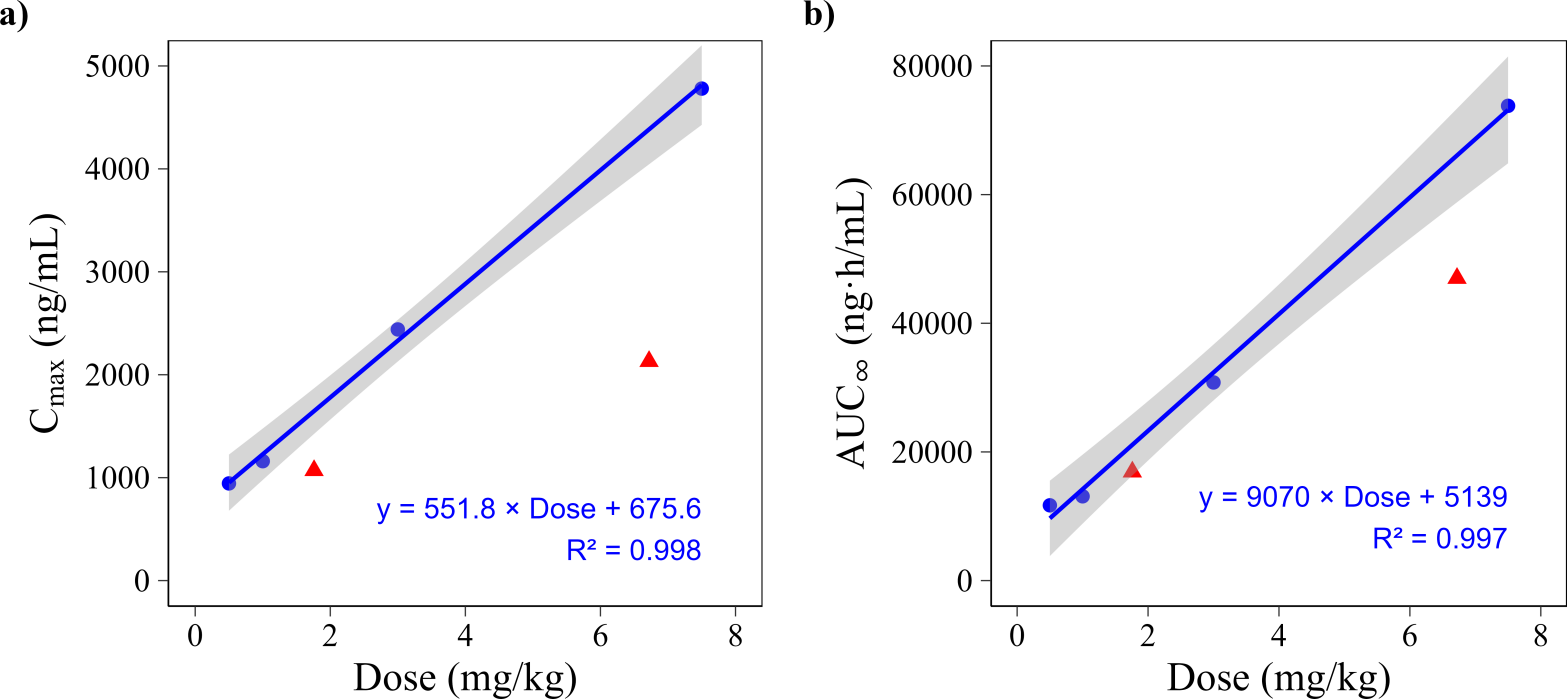
Comparison of oxfendazole exposure following administration of the suspension (blue) in Caucasian and tablet formulation (red) in African healthy adults. (**a**) *C*_max_ and (**b**) AUC_∞_ as a function of dose (mg/kg). The regression line was fitted to suspension data up to 7.5 mg/kg (blue line, shaded area = 95% CI) to estimate expected exposure at equivalent doses. Observed tablet data were overlaid for comparison but were not included in the model fitting.

To contextualize systemic oxfendazole exposure after tablet administration, *C*_max_ and AUC_∞_ values were compared with historical NCA data from Caucasian volunteers who received an oral oxfendazole suspension (SAD study; [9]). A linear regression model was fitted to suspension data for doses ≤7.5 mg/kg to focus on the linear dose range and ensure comparability with the tablet dose levels used in the present study. Tablet doses of 100 and 400 mg were converted to mg/kg using the median body weight in each cohort (56.9 and 59.5 kg), resulting in estimated doses of 1.76 mg/kg and 6.72 mg/kg. Predicted exposure values derived from the regression model were then compared to observed tablet exposure (Fig. 5).

At equivalent doses, exposure following tablet administration in African adults was consistently lower than that of the suspension formulation administered to Caucasian adults. Specifically, *C*_max_ values were approximately 1.5-fold lower at 100 mg and 2.1-fold lower at 400 mg, while AUC_∞_ values were 1.25-fold and 1.41-fold lower, respectively. These differences may reflect both formulation-related factors and population-specific characteristics. Detailed numerical comparisons and plots across the full dose range are provided in Information S8.

### Safety and tolerability

All participants were included in the safety analyses. No AEs of any kind (treatment-emergent or unrelated) were reported in either the active treatment or placebo groups. Special attention was given to laboratory monitoring for risks commonly associated with benzimidazoles, such as mild to moderate liver enzyme elevations, but no such issues were observed. Preventive measures to minimize the risk of pregnancy were implemented to address potential teratogenicity. There were no clinically significant abnormalities in laboratory parameters, vital signs, or physical examinations. No consistent trends over time were noted in mean biochemistry, hematology, or urine dipstick values, and non-clinically significant abnormalities compared to baseline occurred at similar frequencies in the oxfendazole cohorts and the pooled placebo group. Exceptions included low prothrombin time, which was more frequent in cohorts 2 and 3; high aPTT, which was more common in cohort 3; and low bicarbonate, which was more frequently observed in cohorts 1 and 2 and the placebo group (Tables S9 to S11).

Electrocardiographic assessments were conducted through central reading to evaluate the cardiac safety of oxfendazole. A summary of the categorical analysis for all investigated ECG parameters is provided in Table 3. No clinically significant changes in HR, PR or QRS intervals or QTcF were observed. The most frequently recorded non-clinically significant abnormality was a relative change in HR >25% from baseline, recorded in five participants (63%) in cohort 3 and 1 participant (13%) in cohort 1. No such changes were observed in cohort 2 and the placebo group. Notably, four out of five cases of ΔHR >25% in cohort 3 occurred on Day 5, and all were increases in HR. All other abnormal values were incidental, that is, occurred in only one participant. In the central tendency analysis, a mean maximal placebo-corrected change in HR (ΔΔHR) of 6.8 bpm was observed in cohort 1 on Day 1, 3 h after administration. Effects on ΔΔHR were minimal in cohorts 2 and 3.

**TABLE 3:** ECG categorical analysis^*a*^

Category	Cohort 1 100 mg OXF (*N* = 8)	Cohort 2 400 mg OXF (*N* = 8)	Cohort 3, 400 mg OXF, 5 days (*N* = 8)	Placebo pooled (*N* = 6)
ECG mean HR (beats/min)				
HR <40 or >120 bpm	0 (0%)	0 (0%)	0 (0%)	0 (0%)
Δ_Rel_HR >25%	1 (13%)	0 (0%)	5 (63%)	0 (0%)
PR interval aggregate (msec)				
PR Interval >220 msec	1 (13%)	0 (0%)	0 (0%)	0 (0%)
Δ_Rel_PR interval >25%	0 (0%)	0 (0%)	0 (0%)	0 (0%)
QRS interval aggregate (msec)				
QRS interval >120 msec	0 (0%)	0 (0%)	0 (0%)	0 (0%)
Δ_Rel_QRS interval >25%	0 (0%)	0 (0%)	0 (0%)	0 (0%)
QTcF, aggregate (msec)				
QTcF > 450 msec	0 (0%)	0 (0%)	0 (0%)	0 (0%)
ΔQTcF >30 msec	0 (0%)	0 (0%)	0 (0%)	0 (0%)

^*a*^ Data are shown as number of participants (%). ECG, electrocardiogram; *N*, number of participants by treatment group; OXF, oxfendazole. Δ, Change from baseline (time point value – baseline value). Δ_Rel_, Relative change from baseline (100 * [time point value - baseline value]/baseline value). HR, heart rate. QTcF, QT interval corrected according to Fridericia’s formula. Note: for cohort 3, ECGs were also recorded on Day 5 (pre-dose, 1, 2, and 3 h).

QTcF changes from baseline were ≤30 msec, and absolute QTcF values remained ≤450 msec for all participants after administration of oxfendazole. The upper bound of the 90% confidence interval (CI) for ΔΔQTcF exceeded the regulatory threshold of concern (10 ms) at nearly all time points, although this was relative to placebo and did not indicate a consistent QT-prolonging effect.

## DISCUSSION

Oxfendazole is a benzimidazole anthelmintic that has shown efficacy against a broad range of parasitic infections in animals. As a proven veterinary medicine, it holds significant potential to also address unmet treatment needs in multiple human helminth infections. Repurposing veterinary drugs for human use is a cost-effective strategy, as many aspects of their development are already well-established. This can potentially accelerate regulatory approvals and reduce development costs. If proven effective and safe, oxfendazole could become an important addition to the limited therapeutic arsenal against human nematode infections, addressing significant global health challenges.

Oxfendazole has been well tolerated in several mammals and recently demonstrated a favorable PK and safety profile in healthy Caucasian volunteers receiving a liquid formulation. To advance its clinical development, we evaluated a new, field-applicable tablet formulation in healthy adults in Tanzania—a population more representative of the intended target population. This study provides the first evaluation of the tablet formulation in an African setting, confirming its favorable PK and safety profile. The absence of AEs and attainment of pharmacologically relevant plasma exposure supports further clinical development.

### Pharmacokinetics

The PK profile of oxfendazole was broadly consistent with previous findings, showing less than dose-proportional increases in systemic exposure. This non-linear PK has been attributed to solubility-limited absorption, consistent with oxfendazole’s classification as a Biopharmaceutics Classification System Class II compound (low solubility, high permeability).

Absorption from the immediate-release tablet formulation was slightly slower than from the oral suspension used in earlier studies (9, 10) and exposure levels (*C*_max_ and AUC_∞_) appeared lower (Information S8). The most plausible explanation is formulation-related, as the tablet may have slower dissolution and reduced absorption compared with the liquid formulation. However, direct comparisons are limited by differences in study populations and dose levels. The earlier studies were conducted in Caucasian volunteers, whereas this trial was performed in African adults. Population differences in (gut) physiology or genetic polymorphisms in drug-metabolizing enzymes may contribute to the observed differences in exposure.

Oxfendazole undergoes biotransformation through sulfur oxidation to oxfendazole sulfone and sulfur reduction to fenbendazole. The oxidative pathway from fenbendazole to oxfendazole has been well characterized and involves flavin-monooxygenase (FMO) and CYP3A4 enzymes (Virkel 2025; [5, 12]), whereas the specific enzyme(s) mediating the reverse conversion remain incompletely defined. Analogies with the structurally related albendazole support the involvement of FMO3 and CYP3A4 in mediating the sulfoxidation of benzimidazoles (9; Rawden 2000). Inter-individual variability in exposure may be influenced by genetic polymorphisms in these metabolic pathways (12–14).

Moreover, participants in our study were fasted, whereas previous studies also investigated fed states. Indeed, food intake (e.g., a fatty breakfast) has been shown to increase oxfendazole exposure (*C*_max_, AUC) and delay *T*_max_. However, evaluating the impact of food on the new tablet formulation will be important for future clinical development and dose optimization.

Given these multiple, overlapping sources of variability, physiologically based PK modeling could be a valuable tool to disentangle formulation- and population-related effects, inform dose optimization, and support translation to patient populations.

The terminal *t*_1/2_ of oxfendazole was approximately 13 h and appeared to be independent of dose. Although slightly shorter half-lives were reported in previous phase one studies, the differences were small. Inter-individual variability in PK parameters was high, particularly after single-dose administration. While variability appeared lower following multiple dosing, this observation should be interpreted with caution due to the small sample size (*N* = 8) and the absence of extreme outliers, as observed in the 400 mg single-dose cohort.

Metabolite plasma concentrations were generally lower than those of oxfendazole, reflecting relatively minor metabolite formation. The main metabolite was oxfendazole sulfone, consistent with earlier studies. Despite greater accumulation of metabolites, oxfendazole remained the predominant moiety in plasma and is likely the main driver of antiparasitic activity. Notably, despite high variability, trough levels of oxfendazole exceeded efficacious exposures established in preclinical models, supporting the suitability of the tablet formulation for early proof-of-concept trials.

### Safety and tolerability

The oxfendazole tablet formulation was well tolerated at all tested doses—100 mg, 400 mg (single dose), and 400 mg once daily for 5 days—with no AEs reported. This is consistent with previous SAD and MAD studies, which demonstrated a favorable safety profile (9, 10). In those studies, AEs were mild to moderate, with no serious or severe AEs or discontinuations. Headache was the most common treatment-emergent AE in the MAD study. A mild decrease in hemoglobin was observed; however, median levels remained within the normal range, even at a dose of 60 mg/kg once daily, as supported by simulations (15).

The absence of AEs in the present study may reflect the lower dose range tested, the small sample size, and possibly differences between formulations. No clinically significant abnormalities were observed in laboratory parameters, vital signs, physical examinations, and ECG safety parameters.

Although some non-clinically significant laboratory abnormalities compared to baseline were recorded, their frequency appeared similar between oxfendazole and placebo groups. Exceptions included low prothrombin time and elevated aPTT, which were more frequent in the higher dose cohorts and may warrant closer monitoring. Low bicarbonate was more frequent in cohorts 1 and 2 and the placebo group and is therefore unlikely to be treatment-related.

Central tendency analysis suggested that oxfendazole may slightly increase HR. While considered not clinically significant, a positive chronotropic effect cannot be excluded based on the results of this study and warrants surveillance in future clinical trials. No effects on other ECG parameters or cardiac morphological abnormalities were noted. Within the limitations of this study—i.e., the absence of a positive QTc-prolonging control, small participant numbers, and evaluation of only two dose levels—the results do not indicate a QTc-prolonging effect of oxfendazole.

### Strengths and limitations

A major strength of this study is its setting in sub-Saharan Africa, where helminth infections are endemic and future clinical use of oxfendazole is envisioned. Most early-phase clinical trials are conducted in high-income countries, which may limit generalizability. By characterizing oxfendazole’s PK in an African population, this study contributes to the growing body of evidence supporting its clinical development in a global setting. This is particularly relevant given potential regional differences in biochemistry, genetic polymorphisms in drug-metabolizing enzymes, and environmental factors such as diet, microbiome composition, and co-endemic infections. Another strength is the intensive PK sampling, enabling detailed characterization of both oxfendazole and its metabolites.

However, the study had several limitations. Only two cohorts were included in the SAD, which limits the ability to fully assess dose proportionality and complicates comparisons with previous phase 1 studies. Similarly, only one cohort was included in the MAD, which constrains the assessment of accumulation to a single dose level. Nevertheless, the 5-day dosing period provides clear evidence of minimal accumulation of the parent compound, consistent with previously reported MAD data. Meaningful comparisons with previous studies are further limited by differences in formulation and study populations. The relative bioavailability of the tablet formulation could not be formally assessed, as the liquid formulation was not tested in the same population under identical conditions. The relative bioavailability of the tablet formulation could not be formally assessed, as the liquid formulation was not tested in the same population under identical conditions. High inter-individual variability in drug exposure underscores the need for formulation optimization and refined dosing strategies. Advanced technologies, such as enabling formulations, should be explored to improve consistency in exposure. A population PK model is currently being developed to support dose selection in future clinical trials; results will be reported separately.

### Conclusions

The newly developed immediate-release oxfendazole tablet formulation was well tolerated and achieved plasma concentrations previously shown to be efficacious in preclinical models. These findings support continued clinical development and provide a foundation for future proof-of-concept trials to evaluate its efficacy in the treatment of human helminth infections.

## SUPPLEMENTAL MATERIAL

Supplemental material aac.01315-25-s0001.docx Supplemental information and figures.

Supplemental material 2 aac.01315-25-s0002.docx Supplemental table.

ASM does not own the copyrights to Supplemental Material that may be linked to, or accessed through, an article. The authors have granted ASM a non-exclusive, world-wide license to publish the Supplemental Material files. Please contact the corresponding author directly for reuse.
