# Supplementary material for: Pharmacokinetics, safety, and tolerability of an oxfendazole tablet formulation: a phase 1, randomized, placebo-controlled trial in healthy African volunteers
Source: Antimicrob Agents Chemother. 2026 Feb 18;70(4):e01315-25. doi: 10.1128/aac.01315-25 (PMC13041396; doi:10.1128/aac.01315-25)
Supplement: Supplemental material — Supplemental information and figures. [file aac.01315-25-s0001.docx]

**Supporting information**

**Pharmacokinetics, safety, and tolerability of an oxfendazole tablet formulation: a phase 1, randomized, placebo-controlled trial in healthy African volunteers**

Contents

[S1 Investigational Product 2](#_Toc196177319)

[S2 Sentinel design and dose escalation steps 3](#_Toc196177320)

[S3 Schedule of events 4](#_Toc196177321)

[S4 HPLC-MS/MS conditions 7](#_Toc196177322)

[S5 Individual plasma concentration–time profiles, semi-logarithmic scale 8](#_Toc196177323)

[S6 Arithmetic mean plasma concentration–time profiles 10](#_Toc196177324)

[S7 Dose-normalized exposure 11](#_Toc196177325)

[S8 Evaluation of exposure differences between tablet and liquid formulation 13](#_Toc196177326)

S1 Investigational product

Oxfendazole was supplied as an immediate-release tablet formulation in aluminum/aluminum blister packs. Each tablet, manufactured by Syngene (Bengaluru, India), contained 100 mg of oxfendazole (40% w/w) and the following excipients: microcrystalline cellulose (55.7% w/w), sodium starch glycolate (3.5% w/w) and magnesium stearate (0.8% w/w). The dissolution profile of the tablet formulation closely resembled that of the Synanthic® suspension used in the previous FIH study {Bach, 2020 #3419}. Specifically, more than 80% and more than 90% of the oxfendazole were released within 30 minutes from the tablet formulation and the Synanthic® suspension, respectively (USP II dissolution test). All dissolution results were consistent with an immediate-release product. A visually matching placebo contained the same excipients. Study drugs were stored at room temperature below 30°C.

S2 Sentinel design and dose escalation steps


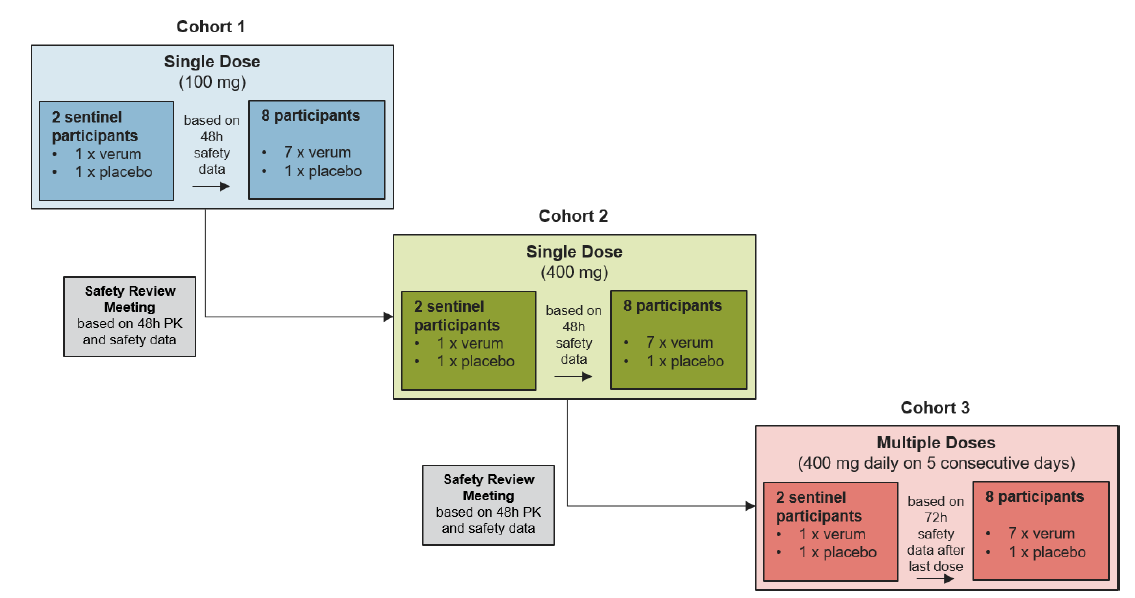


**Fig A. Sentinel design and dose escalation steps.**

The study included three sequential cohorts: two single-dose cohorts (100 mg and 400 mg) and one multiple-dose cohort (400 mg daily for 5 days). Each cohort began with two sentinel participants (1 oxfendazole, 1 placebo), followed by a safety surveillance of 48 h (cohort 1 and cohort 2) or 72 hours (cohort 3) and the Investigator’s evaluation before enrolling the remaining participants. Dose escalation to the next cohort was permitted only after the Safety Review Committee (SRC) confirmed that the preceding dose was safe and well tolerated.

S3 Schedule of events


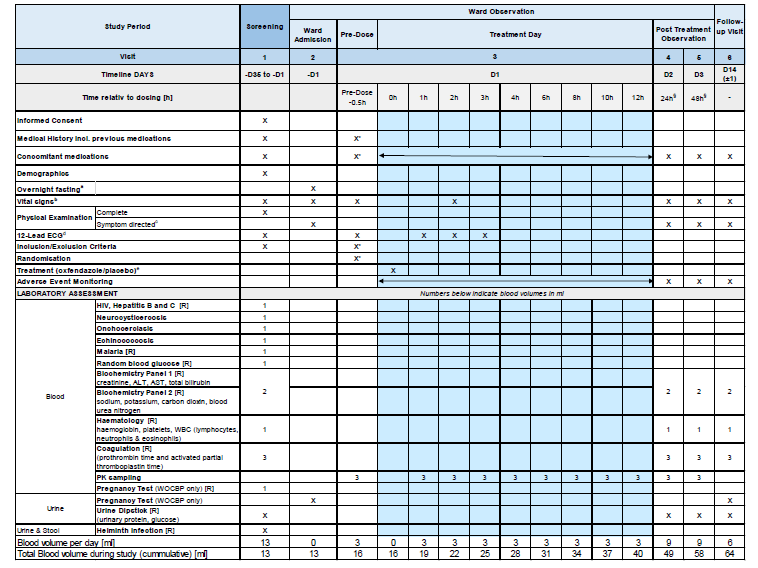


**Fig A.** **Schedule of events for Cohorts 1 and 2.**

^a^On Day-1, participants need to fasten overnight for at least 8 hours prior to the receipt of the study treatment on the following Day 1.

^b^BP and HR are measured after 10 min rest in a supine position.

^c^Symptom directed examination is done once per day on specified days (time point not specified)

^d^Three repeat ECGs with at least 1 minute apart will be done. Healthy volunteers should rest in the supine position for 10 minutes before ECG measurements.

At screening, the ECGs will be reviewed by a study clinician and a second review will be done by a senior study clinician. If needed a cardiologist will be consulted. ECGs at pre-dose and at time points 1h, 2h and 3h on Day 1 will be reviewed centrally.

^e^Participants will remain fasting for 2 hours after administration of study treatment.

R. Repeat if done > 7 days prior to D1.

* These activities/assessments can be done at any time on D1 prior to study treatment (no specific time window). Only PK sampling needs to be done at time point indicated as -0.5h prior to treatment. ECG and vital signs need to be done very close to -0.5h (prior to PK sampling).

§ PK sampling needs to be done on nominal time (24h and 48h post dose) within the allowed time window (Table 2). All other assessments like concomitant medication, vital signs, symptom directed physical examination, AE monitoring, safety blood withdrawal (biochemistry, haematology, coagulation) and urine sampling can be done at any time during the Day.


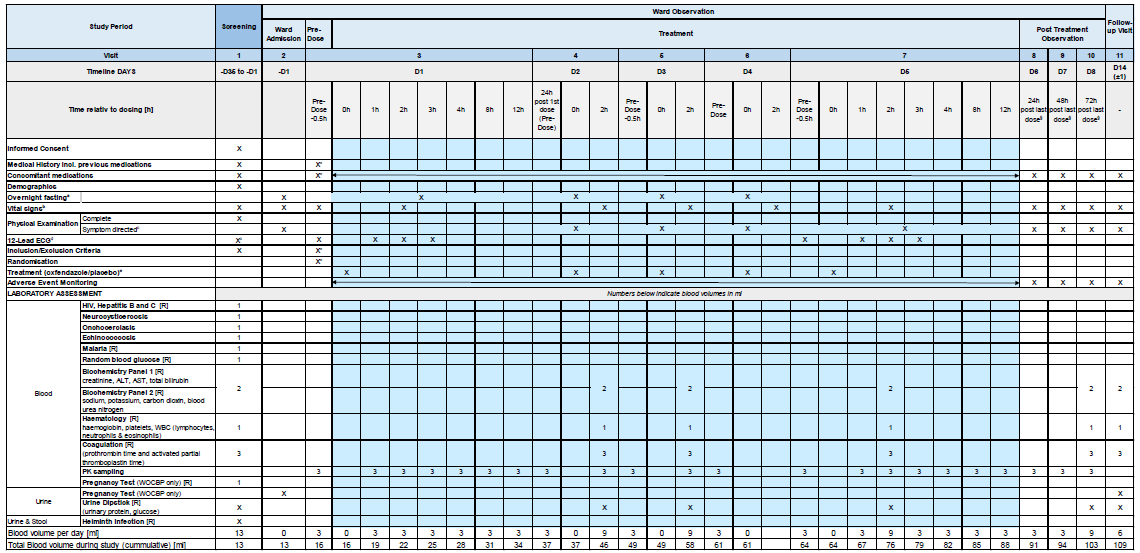


**Fig B. Schedule of events for Cohort 3.**

^a^Participants need to fast overnight for at least 8 hours prior to the receipt of the study treatment on the following days:

D-1: Fasting at least 8h overnight prior to dosage on D1

D1: Fasting at least 8h overnight prior to dosage on D2

D2: Fasting at least 8h overnight prior to dosage on D3

D3: Fasting at least 8h overnight prior to dosage on D4

D4: Fasting at least 8h overnight prior to dosage on D5

^b^BP and HR are measured after 10 min rest in a supine position.

^c^Symptom directed examination is done once per day on specified days (time point not specified)

^d^Three repeat ECGs with at least 1 minute apart will be done. Healthy volunteers should rest in the supine position for 10 minutes before ECG measurements.

At screening, the ECGs will be reviewed by a study clinician and a second review will be done by a senior study clinician. If needed a cardiologist will be consulted. ECGs at pre-dose and at time points 1h, 2h and 3h on Day 1 and 5 will be reviewed centrally.

^e^Participants will remain fasting for 2 hours after administration of study treatment.

R. Repeat if done > 7 days prior to D1.

* These activities/assessments can be done at any time on D1 prior to study treatment (no specific time window). Only PK sampling needs to be done at time point indicated as -0.5h prior to treatment. ECG and vital signs need to be done very close to -0.5h (prior to PK sampling).

§ PK sampling needs to be done on nominal time (24h and 72h post last dose) within the allowed time window (Table 4). All other assessments like concomitant medication, vital signs, symptom directed physical examination, AE monitoring, safety blood withdrawal (biochemistry, haematology, coagulation) and urine sampling can be done at any time during the Day.

S4 HPLC-MS/MS conditions

Plasma concentrations of oxfendazole, fenbendazole, and fenbendazole sulfone were quantified using a validated liquid chromatography tandem mass spectrometry HPLC-MS/MS) method.

Analysis was carried out using a TSQ Quantum Access mass spectrometer (Thermo Fisher Scientific, San Jose, CA, USA) coupled to an Agilent 1200 HPLC pump (Agilent Technologies Inc, Santa Clara, CA, USA). This system was fitted with a CTC PAL Autosampler (CTC Analytics AG, Zwingen, Switzerland) and a column oven (HotDog 5090, Prolab GmbH, Reinach, Switzerland). Deuterated analogues of analytes were used as internal standards. Plasma was precipitated with internal standards. The mixture was vortex-mixed and centrifuged, and 5 µL of the supernatant injected for drug quantification.

A gradient elution of analytes was carried out at 40 °C in a mixture of mobile phases comprising 0.5 % formic acid in water (A) and 0.2 % formic acid in methanol (B) at a constant flow rate of 0.5 mL/min, using a C_18_  column (Hypersil Gold, 2.1 x 50 mm, 3 µm, Thermo Fisher Scientific Inc., Waltham, MA, USA). The mobile phase gradient was applied in a sequence of 95 % of mobile phase A between 0.00 min and 0.20 min, 2 % of mobile phase A between 1.50 min and 3.55 min, and then 95 % of mobile phase A between 3.60 min and 4.00 min. Inter-assay imprecision, expressed as coefficient of variation, was between 3.4 % and 15.2 %, and inter-assay accuracy was between 90.5 % and 105.1%.

Analyte recovery was between 95.2 % and 105.1%. The lower limits of quantification (LLOQ) were 2 ng/mL for oxfendazole, 1 ng/mL for fenbendazole and 1 ng/mL for fenbendazole sulfone.

S5 Individual plasma concentration–time profiles, semi-logarithmic scale


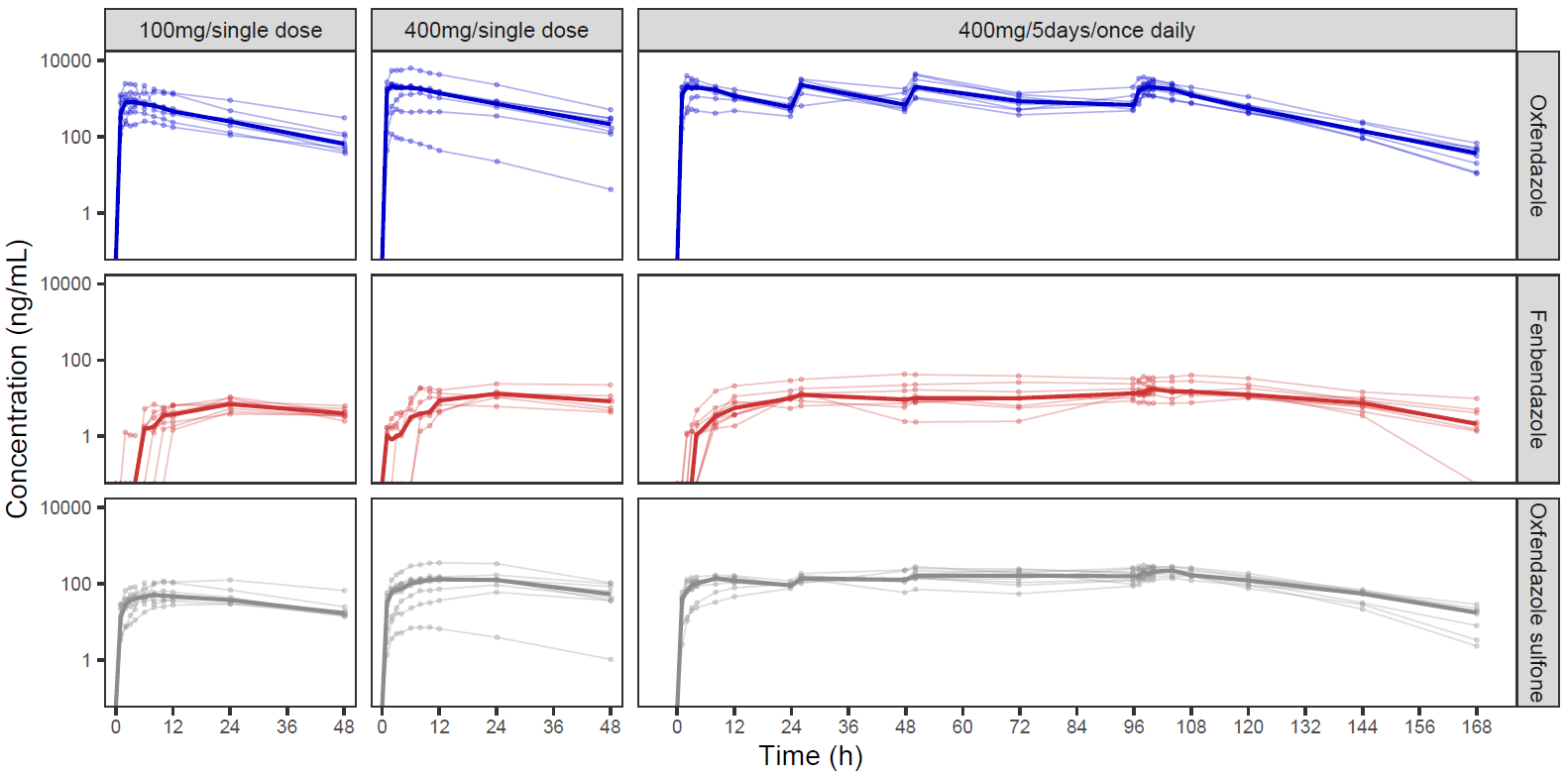


**Fig. A. Plasma concentration–time profiles on a semi-logarithmic scale.**

Individual plasma concentration–time profiles of oxfendazole and its metabolites, fenbendazole and oxfendazole sulfone, in healthy adult subjects (n = 8 per cohort) following administration of oxfendazole as a single 100 mg dose (Cohort 1), single 400 mg dose (Cohort 2), or 400 mg once daily for 5 days (Cohort 3). Thin lines represent individual subject profiles; bold lines represent the median concentration at each time point. For fenbendazole, all pharmacokinetic samples from one subject in Cohort 2 were below the limit of quantification and are not shown.


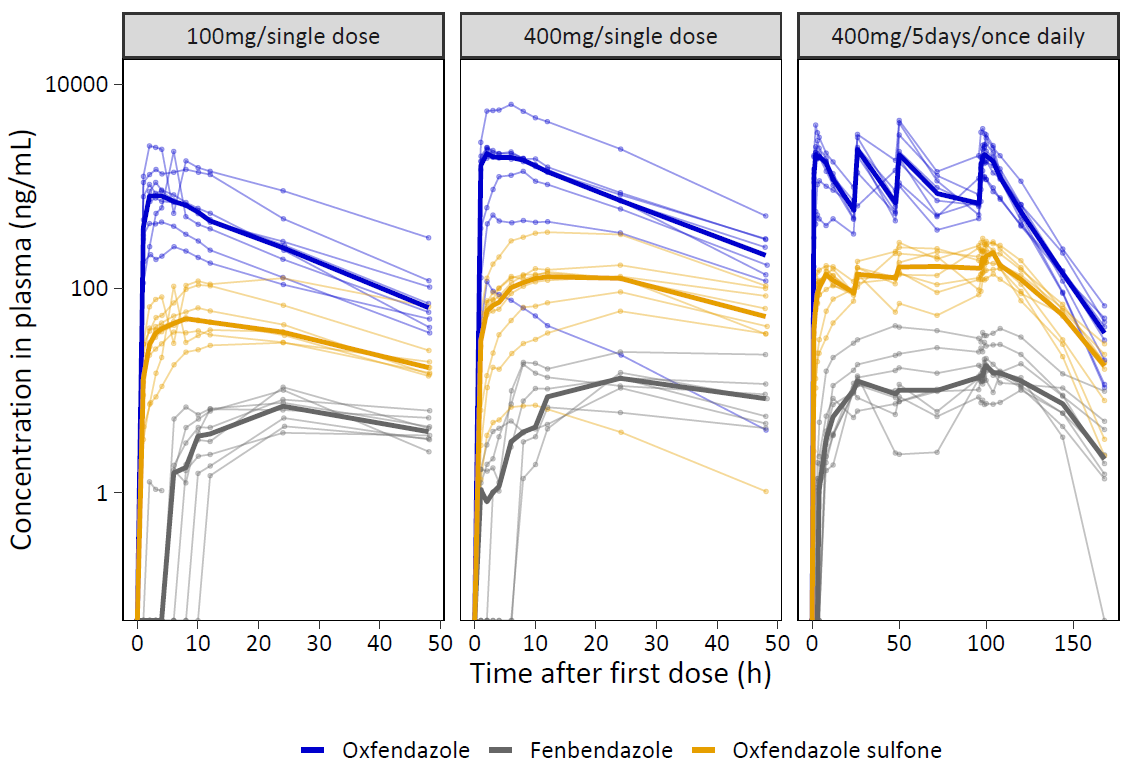


S6 Arithmetic mean plasma concentration–time profiles


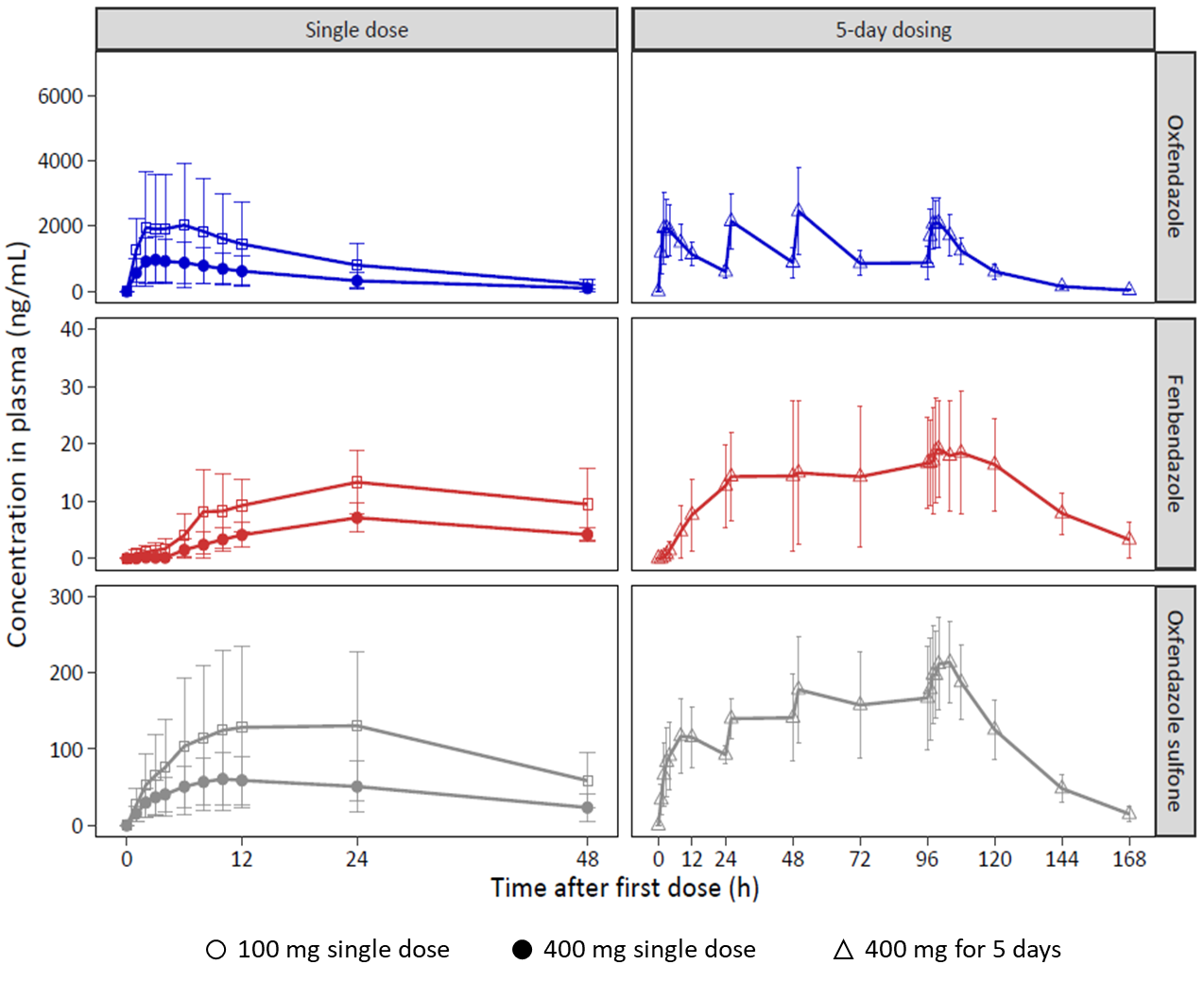


**Fig. A. Mean plasma concentration–time profiles on a semi-logarithmic scale.**

Mean plasma concentration versus time profiles of oxfendazole and its metabolites, fenbendazole and oxfendazole sulfone in healthy adult subjects (n=8 per cohort) following administration of oxfendazole as a single 100 mg dose (Cohort 1), single 400 mg dose (Cohort 2), or 400 mg once daily for 5 days (Cohort 3).

Arithmetic mean concentrations at each nominal blood sampling time point are shown, along with the corresponding standard deviations. For fenbendazole, all pharmacokinetic samples from one subject in cohort 2 were below the limit of quantification and are not shown.

S7 Dose-normalized exposure


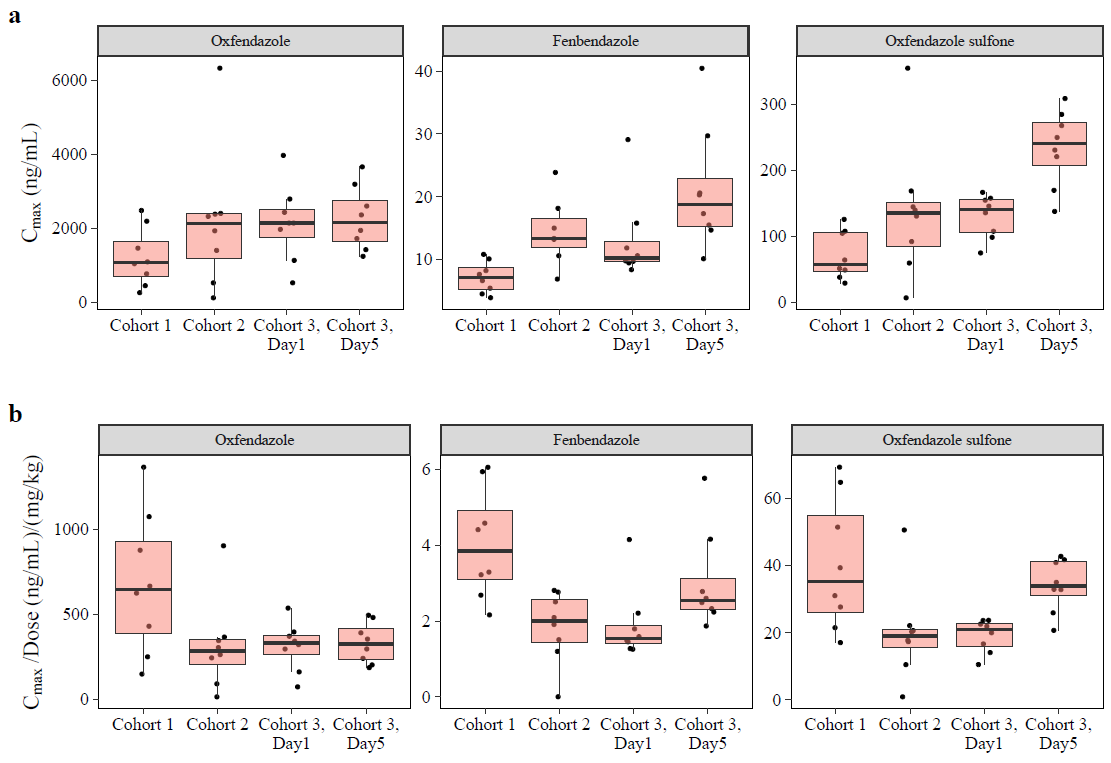


**Fig. A. Peak plasma concentration (C_max_) of oxfendazole, fenbendazole, and oxfendazole sulfone across dosing cohorts.**

**a)** absolute C_max_, and **b)** dose-normalized C_max_. Cohort 1: 100 mg oxfendazole single dose; Cohort 2: 400 mg single dose; Cohort 3: 400 mg once daily for 5 days. Dose normalization was based on dose per kg body weight for each participant. Boxes represent the interquartile range (IQR), the bold line indicates the median, and whiskers extend to 1.5× IQR. Individual participant values are shown as dots (N=8 per cohort). For fenbendazole, all pharmacokinetic samples from one subject in Cohort 2 were below the lower limit of quantification (LLOQ) and are not shown (N = 7 for fenbendazole in cohort 2). Measurements are shown for Cohorts 1–3, with Cohort 3 sampled on both Day 1 and Day 5; Day 5 exposures reflect drug accumulation.

**
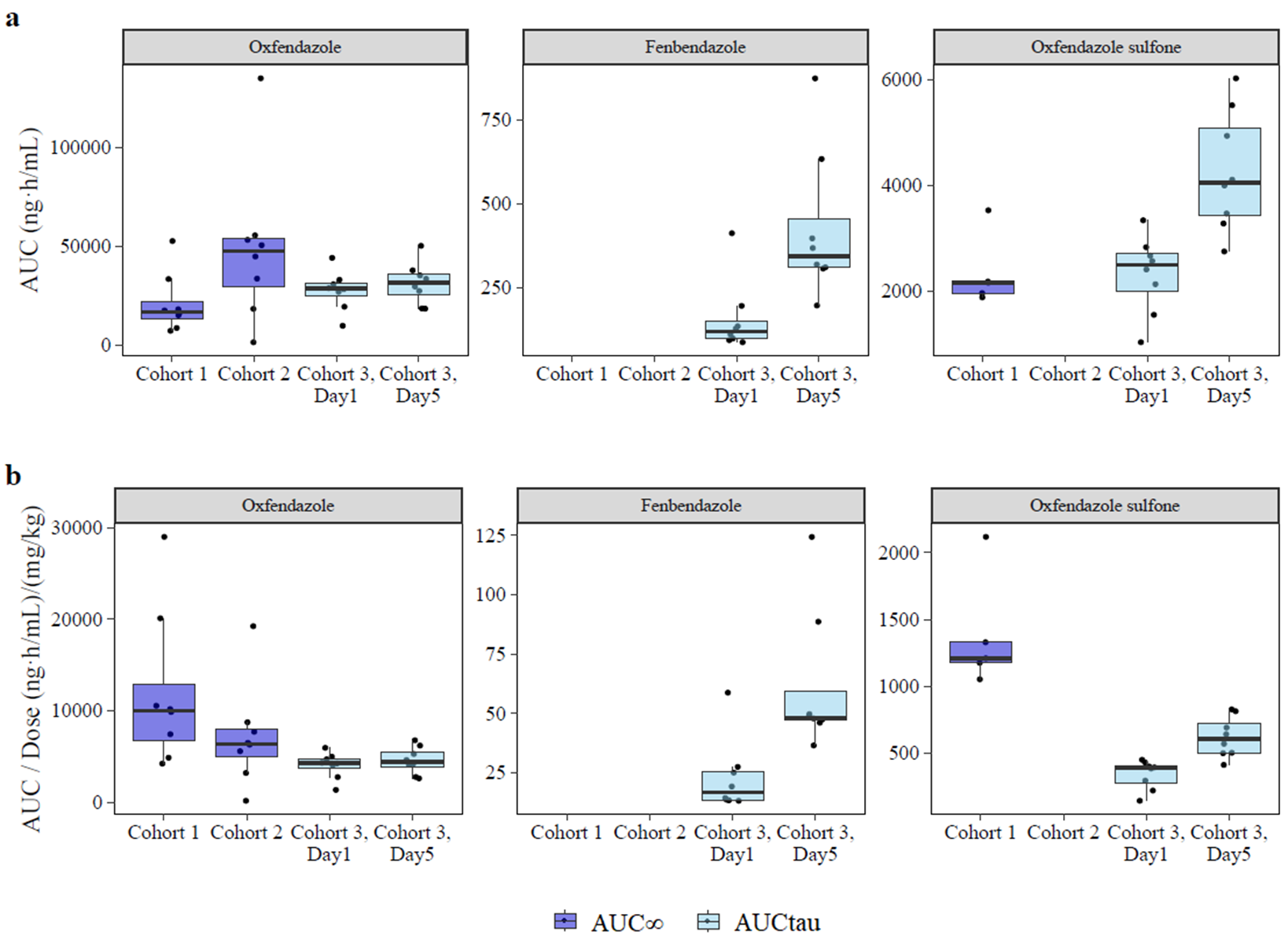
**

**Fig. B. Area under the plasma concentration–time curve (AUC) of oxfendazole, fenbendazole, and oxfendazole sulfone across dosing cohorts.**

**a)** absolute AUC, and **b)** dose-normalized AUC. Cohort 1: 100 mg oxfendazole single dose; Cohort 2: 400 mg single dose; Cohort 3: 400 mg once daily for 5 days. Dose normalization was based on dose per kg body weight. AUC values are stratified by calculation method: AUC_∞_ (from time zero to infinity) for single-dose cohorts, and AUC_tau_ (during a 24-hour dosing interval) for the multiple-dose cohort.   Boxes represent the interquartile range (IQR), the bold line indicates the median, and whiskers extend to 1.5× IQR. Individual participant values are shown as dots (N=8 per cohort). However, AUC was not estimated (NE) for fenbendazole in Cohorts 1 and 2, and for oxfendazole sulfone in Cohort 2, due to insufficient data (N < 3). For fenbendazole, all pharmacokinetic samples from one subject in cohort 2 were below the lower limit of quantification (LLOQ) and are not shown (N = 7 for fenbendazole in cohort 2). AUC_∞_ for oxfendazole sulfone in Cohort 1 was estimated based on N = 5 participants. Measurements are shown for Cohorts 1–3, with Cohort 3 sampled on both Day 1 and Day 5; Day 5 exposures reflect drug accumulation.

S8 Evaluation of exposure differences between tablet and liquid formulation

To evaluate relative bioavailability, we analyzed exposure data from the previously published single ascending dose (SAD) study[1], which reported exposure for an oxfendazole liquid formulation (non-compartmental analysis).

A linear regression model was fitted to C_max_ values observed at doses ≤ 7.5 mg/kg, restricting the fit to the linear range to ensure comparability with the dose levels tested for the tablet formulation. For the tablet data, the administered doses (100 mg and 400 mg) were converted to mg/kg using the median body weight reported in each cohort (56.9 kg and 59.5 kg, respectively), resulting in estimated doses of 1.76 mg/kg and 6.72 mg/kg. The regression model from the solution data was used to predict C_max_ at these doses. Predicted values were compared to the observed tablet Cmax, and fold differences were calculated to quantify the relative reduction in systemic exposure associated with the tablet formulation.

The same approach was applied to AUC_∞_.

Exposure following tablet administration was consistently lower than that of the liquid formulation after a single dose of oxfendazole (**Table A**, **Figure A** and **B**).

Similar trends were observed when comparing multiple-dose data (not shown) [2], with lower exposure consistently seen following administration of the tablet formulation.

It is important to note that the study populations differed: the liquid formulation was administered to healthy White volunteers, whereas the tablet formulation was tested in healthy adults from Tanzania. Therefore, differences in systemic exposure may reflect not only formulation-related factors but also population-specific characteristics.

**Table A.** Comparison of observed and predicted exposure for the oxfendazole tablet formulation.

| **Dose**  **(mg)** | **Dose (mg/kg)** | **C_max_** | | |  | **AUC_∞_** | | |
| --- | --- | --- | --- | --- | --- | --- | --- | --- |
|  |  | **Observed (ng/mL)** | **Predicted (ng/mL)** | **Fold difference** |  | **Observed (ng·h/mL)** | **Predicted (ng·h/mL)** | **Fold difference** |
| 100 | 1.76 | 1070 | 1647 | 1.54 |  | 16900 | 21103 | 1.25 |
| 400 | 6.72 | 2130 | 4384 | 2.06 |  | 47000 | 6609 | 1.41 |

values were derived using a regression model based on the liquid formulation, assuming that the same dose (mg/kg) would result in equivalent exposure. Observed values are reported as medians.

**
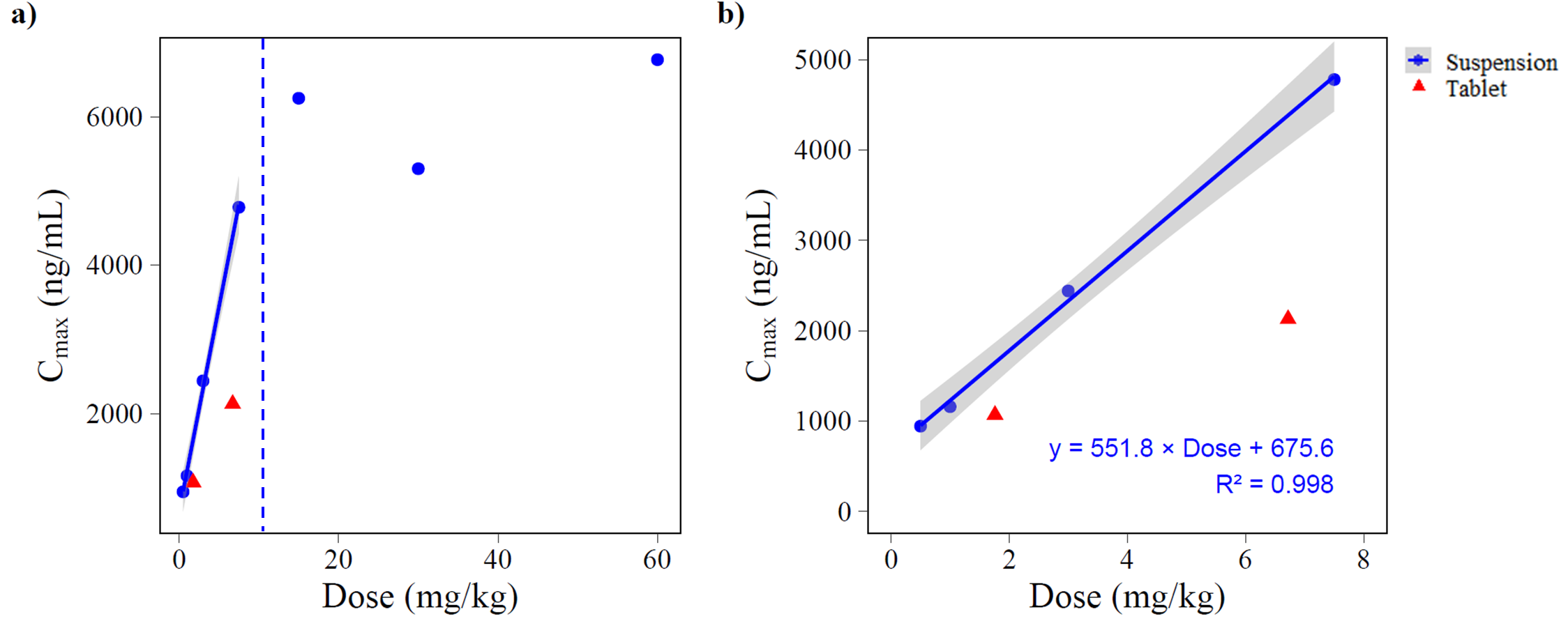
**

**Fig. A.** Comparison of C_max_ following administration of oxfendazole solution and tablet formulations.

**a)** Full dataset showing observed C_max_ values for the solution (blue) and tablet (red) formulations across the dose range, with a vertical dashed line at 7.5 mg/kg indicating the upper limit of data included in the regression model. **b)** Zoomed-in view with linear regression based on solution data up to 7.5 mg/kg. The equation and R² are shown. Tablet data are overlaid for comparison but were not included in the model fitting.

**
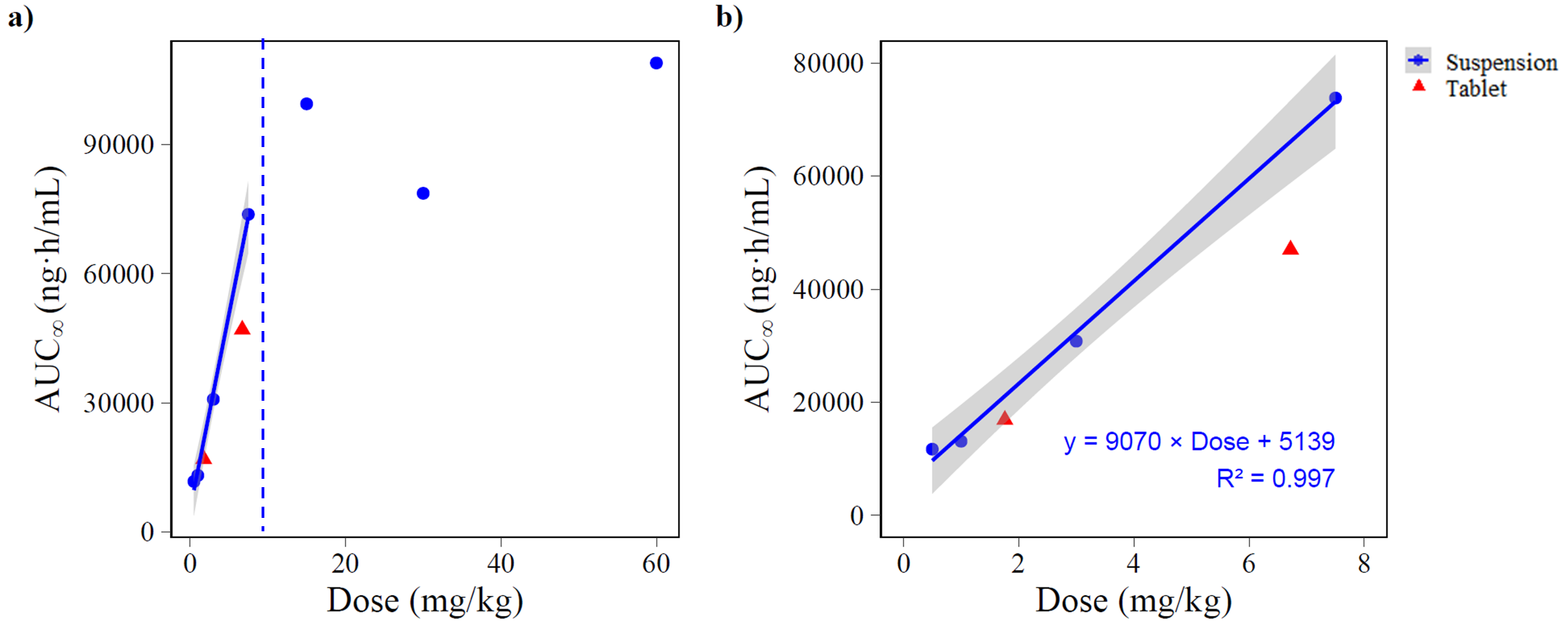
**

**Fig. B.** Comparison of AUC_∞_ following administration of oxfendazole solution and tablet formulations.

Panels and analysis as described for Fig. A.

**References:**

[1] An G, Murry DJ, Gajurel K, Bach T, Deye G, Stebounova LV, et al. Pharmacokinetics, Safety, and Tolerability of Oxfendazole in Healthy Volunteers: a Randomized, Placebo-Controlled First-in-Human Single-Dose Escalation Study. Antimicrobial agents and chemotherapy. 2019;63(4).

[2] Bach T, Galbiati S, Kennedy JK, Deye G, Nomicos EYH, Codd EE, et al. Pharmacokinetics, Safety, and Tolerability of Oxfendazole in Healthy Adults in an Open-Label Phase 1 Multiple Ascending Dose and Food Effect Study. Antimicrobial agents and chemotherapy. 2020;64(11).
