# Supplementary material for: Pharmacokinetics, safety, and tolerability of an oxfendazole tablet formulation: a phase 1, randomized, placebo-controlled trial in healthy African volunteers
Source: Antimicrob Agents Chemother. 2026 Feb 18;70(4):e01315-25. doi: 10.1128/aac.01315-25 (PMC13041396; doi:10.1128/aac.01315-25)
Supplement: Supplemental material 2 — Supplemental table. [file aac.01315-25-s0002.docx]

**Supporting information S9-11**

**Pharmacokinetics, safety, and tolerability of an oxfendazole tablet formulation: a phase 1, randomized, placebo-controlled trial in healthy African volunteers**

Contents

[S9 Summay of non-clinically significant, abnormal biochemical results 2](#_Toc196331038)

[S10 Summary of non-clinically significant, abnormal hematology results 21](#_Toc196331039)

[S11 Summary of non-clinically significant, abnormal coagulation results 36](#_Toc196331040)

S9 Summary of non-clinically significant, abnormal biochemical results

| Laboratory test (units) | Visit | Treatment  received |  |  |  | Result |  |  |
| --- | --- | --- | --- | --- | --- | --- | --- | --- |
|  |  |  | n expected | Missing, n (%) | Below reference but not clinically significant | Below reference and clinically significant | Above reference but not clinically significant | Above reference and clinically significant |
| Sodium (mmol/L) | Baseline | Oxfendazole Cohort 1 | 8 | 0 (0.0) | 0 (0.0%) | 0 (0.0%) | 0 (0.0%) | 0 (0.0%) |
|  |  | Oxfendazole Cohort 2 | 8 | 0 (0.0) | 0 (0.0%) | 0 (0.0%) | 0 (0.0%) | 0 (0.0%) |
|  |  | Oxfendazole Cohort 3 | 8 | 0 (0.0) | 3 (37.5%) | 0 (0.0%) | 0 (0.0%) | 0 (0.0%) |
|  |  | Pooled placebo | 6 | 0 (0.0) | 1 (16.7%) | 0 (0.0%) | 0 (0.0%) | 0 (0.0%) |
|  | D2 | Oxfendazole Cohort 1 | 8 | 0 (0.0) | 0 (0.0%) | 0 (0.0%) | 0 (0.0%) | 0 (0.0%) |
|  |  | Oxfendazole Cohort 2 | 8 | 0 (0.0) | 1 (12.5%) | 0 (0.0%) | 0 (0.0%) | 0 (0.0%) |
|  |  | Oxfendazole Cohort 3 | 8 | 0 (0.0) | 3 (37.5%) | 0 (0.0%) | 0 (0.0%) | 0 (0.0%) |
|  |  | Pooled placebo | 6 | 0 (0.0) | 1 (16.7%) | 0 (0.0%) | 0 (0.0%) | 0 (0.0%) |
|  | D3 | Oxfendazole Cohort 1 | 8 | 0 (0.0) | 1 (12.5%) | 0 (0.0%) | 0 (0.0%) | 0 (0.0%) |
|  |  | Oxfendazole Cohort 2 | 8 | 0 (0.0) | 0 (0.0%) | 0 (0.0%) | 0 (0.0%) | 0 (0.0%) |
|  |  | Oxfendazole Cohort 3 | 8 | 0 (0.0) | 1 (12.5%) | 0 (0.0%) | 0 (0.0%) | 0 (0.0%) |
|  |  | Pooled placebo | 6 | 0 (0.0) | 3 (50.0%) | 0 (0.0%) | 0 (0.0%) | 0 (0.0%) |

| Laboratory test (units) | Visit | Treatment  received |  |  |  | Result |  |  |
| --- | --- | --- | --- | --- | --- | --- | --- | --- |
|  |  |  | n expected | Missing, n (%) | Below reference but not clinically significant | Below reference and clinically significant | Above reference but not clinically significant | Above reference and clinically significant |
|  | D5 | Oxfendazole Cohort 1 | - | - | - | - | - | - |
|  |  | Oxfendazole Cohort 2 | - | - | - | - | - | - |
|  |  | Oxfendazole Cohort 3 | 8 | 0 (0.0) | 1 (12.5%) | 0 (0.0%) | 0 (0.0%) | 0 (0.0%) |
|  |  | Pooled placebo | 2 | 0 (0.0) | 1 (50.0%) | 0 (0.0%) | 0 (0.0%) | 0 (0.0%) |
|  | D8 | Oxfendazole Cohort 1 | - | - | - | - | - | - |
|  |  | Oxfendazole Cohort 2 | - | - | - | - | - | - |
|  |  | Oxfendazole Cohort 3 | 8 | 0 (0.0) | 0 (0.0%) | 0 (0.0%) | 0 (0.0%) | 0 (0.0%) |
|  |  | Pooled placebo | 2 | 0 (0.0) | 0 (0.0%) | 0 (0.0%) | 0 (0.0%) | 0 (0.0%) |
|  | D14 | Oxfendazole Cohort 1 | 8 | 0 (0.0) | 0 (0.0%) | 0 (0.0%) | 0 (0.0%) | 0 (0.0%) |
|  |  | Oxfendazole Cohort 2 | 8 | 0 (0.0) | 2 (25.0%) | 0 (0.0%) | 0 (0.0%) | 0 (0.0%) |
|  |  | Oxfendazole Cohort 3 | 8 | 0 (0.0) | 2 (25.0%) | 0 (0.0%) | 0 (0.0%) | 0 (0.0%) |
|  |  | Pooled placebo | 6 | 0 (0.0) | 3 (50.0%) | 0 (0.0%) | 0 (0.0%) | 0 (0.0%) |

| Laboratory test (units) | Visit | Treatment  received |  |  |  | Result |  |  |
| --- | --- | --- | --- | --- | --- | --- | --- | --- |
|  |  |  | n expected | Missing, n (%) | Below reference but not clinically significant | Below reference and clinically significant | Above reference but not clinically significant | Above reference and clinically significant |
| Potassium (mmol/L) | Baseline | Oxfendazole Cohort 1 | 8 | 0 (0.0) | 0 (0.0%) | 0 (0.0%) | 0 (0.0%) | 0 (0.0%) |
|  |  | Oxfendazole Cohort 2 | 8 | 0 (0.0) | 0 (0.0%) | 0 (0.0%) | 0 (0.0%) | 0 (0.0%) |
|  |  | Oxfendazole Cohort 3 | 8 | 0 (0.0) | 0 (0.0%) | 0 (0.0%) | 0 (0.0%) | 0 (0.0%) |
|  |  | Pooled placebo | 6 | 0 (0.0) | 0 (0.0%) | 0 (0.0%) | 0 (0.0%) | 0 (0.0%) |
|  | D2 | Oxfendazole Cohort 1 | 8 | 0 (0.0) | 0 (0.0%) | 0 (0.0%) | 0 (0.0%) | 0 (0.0%) |
|  |  | Oxfendazole Cohort 2 | 8 | 0 (0.0) | 1 (12.5%) | 0 (0.0%) | 0 (0.0%) | 0 (0.0%) |
|  |  | Oxfendazole Cohort 3 | 8 | 0 (0.0) | 0 (0.0%) | 0 (0.0%) | 0 (0.0%) | 0 (0.0%) |
|  |  | Pooled placebo | 6 | 0 (0.0) | 0 (0.0%) | 0 (0.0%) | 0 (0.0%) | 0 (0.0%) |
|  | D3 | Oxfendazole Cohort 1 | 8 | 0 (0.0) | 0 (0.0%) | 0 (0.0%) | 0 (0.0%) | 0 (0.0%) |
|  |  | Oxfendazole Cohort 2 | 8 | 0 (0.0) | 0 (0.0%) | 0 (0.0%) | 2 (25.0%) | 0 (0.0%) |
|  |  | Oxfendazole Cohort 3 | 8 | 0 (0.0) | 0 (0.0%) | 0 (0.0%) | 1 (12.5%) | 0 (0.0%) |
|  |  | Pooled placebo | 6 | 0 (0.0) | 0 (0.0%) | 0 (0.0%) | 0 (0.0%) | 0 (0.0%) |

| Laboratory test (units) | Visit | Treatment  received |  |  |  | Result |  |  |
| --- | --- | --- | --- | --- | --- | --- | --- | --- |
|  |  |  | n expected | Missing, n (%) | Below reference but not clinically significant | Below reference and clinically significant | Above reference but not clinically significant | Above reference and clinically significant |
|  | D5 | Oxfendazole Cohort 1 | - | - | - | - | - | - |
|  |  | Oxfendazole Cohort 2 | - | - | - | - | - | - |
|  |  | Oxfendazole Cohort 3 | 8 | 0 (0.0) | 0 (0.0%) | 0 (0.0%) | 0 (0.0%) | 0 (0.0%) |
|  |  | Pooled placebo | 2 | 0 (0.0) | 0 (0.0%) | 0 (0.0%) | 0 (0.0%) | 0 (0.0%) |
|  | D8 | Oxfendazole Cohort 1 | - | - | - | - | - | - |
|  |  | Oxfendazole Cohort 2 | - | - | - | - | - | - |
|  |  | Oxfendazole Cohort 3 | 8 | 0 (0.0) | 0 (0.0%) | 0 (0.0%) | 0 (0.0%) | 0 (0.0%) |
|  |  | Pooled placebo | 2 | 0 (0.0) | 0 (0.0%) | 0 (0.0%) | 0 (0.0%) | 0 (0.0%) |
|  | D14 | Oxfendazole Cohort 1 | 8 | 0 (0.0) | 0 (0.0%) | 0 (0.0%) | 0 (0.0%) | 0 (0.0%) |
|  |  | Oxfendazole Cohort 2 | 8 | 0 (0.0) | 0 (0.0%) | 0 (0.0%) | 0 (0.0%) | 0 (0.0%) |
|  |  | Oxfendazole Cohort 3 | 8 | 0 (0.0) | 0 (0.0%) | 0 (0.0%) | 0 (0.0%) | 0 (0.0%) |
|  |  | Pooled placebo | 6 | 0 (0.0) | 0 (0.0%) | 0 (0.0%) | 0 (0.0%) | 0 (0.0%) |

| Laboratory test (units) | Visit | Treatment  received |  |  |  | Result |  |  |
| --- | --- | --- | --- | --- | --- | --- | --- | --- |
|  |  |  | n expected | Missing, n (%) | Below reference but not clinically significant | Below reference and clinically significant | Above reference but not clinically significant | Above reference and clinically significant |
| Blood urea nitrogen (mmol/L) | Baseline | Oxfendazole Cohort 1 | 8 | 0 (0.0) | 3 (37.5%) | 0 (0.0%) | 0 (0.0%) | 0 (0.0%) |
|  |  | Oxfendazole Cohort 2 | 8 | 0 (0.0) | 4 (50.0%) | 0 (0.0%) | 0 (0.0%) | 0 (0.0%) |
|  |  | Oxfendazole Cohort 3 | 8 | 0 (0.0) | 4 (50.0%) | 0 (0.0%) | 0 (0.0%) | 0 (0.0%) |
|  |  | Pooled placebo | 6 | 0 (0.0) | 1 (16.7%) | 0 (0.0%) | 0 (0.0%) | 0 (0.0%) |
|  | D2 | Oxfendazole Cohort 1 | 8 | 0 (0.0) | 4 (50.0%) | 0 (0.0%) | 0 (0.0%) | 0 (0.0%) |
|  |  | Oxfendazole Cohort 2 | 8 | 0 (0.0) | 1 (12.5%) | 0 (0.0%) | 0 (0.0%) | 0 (0.0%) |
|  |  | Oxfendazole Cohort 3 | 8 | 0 (0.0) | 5 (62.5%) | 0 (0.0%) | 0 (0.0%) | 0 (0.0%) |
|  |  | Pooled placebo | 6 | 0 (0.0) | 0 (0.0%) | 0 (0.0%) | 0 (0.0%) | 0 (0.0%) |
|  | D3 | Oxfendazole Cohort 1 | 8 | 0 (0.0) | 4 (50.0%) | 0 (0.0%) | 0 (0.0%) | 0 (0.0%) |
|  |  | Oxfendazole Cohort 2 | 8 | 0 (0.0) | 0 (0.0%) | 0 (0.0%) | 0 (0.0%) | 0 (0.0%) |
|  |  | Oxfendazole Cohort 3 | 8 | 0 (0.0) | 3 (37.5%) | 0 (0.0%) | 0 (0.0%) | 0 (0.0%) |
|  |  | Pooled placebo | 6 | 0 (0.0) | 0 (0.0%) | 0 (0.0%) | 0 (0.0%) | 0 (0.0%) |

| Laboratory test (units) | Visit | Treatment  received |  |  |  | Result |  |  |
| --- | --- | --- | --- | --- | --- | --- | --- | --- |
|  |  |  | n expected | Missing, n (%) | Below reference but not clinically significant | Below reference and clinically significant | Above reference but not clinically significant | Above reference and clinically significant |
|  | D5 | Oxfendazole Cohort 1 | - | - | - | - | - | - |
|  |  | Oxfendazole Cohort 2 | - | - | - | - | - | - |
|  |  | Oxfendazole Cohort 3 | 8 | 0 (0.0) | 0 (0.0%) | 0 (0.0%) | 0 (0.0%) | 0 (0.0%) |
|  |  | Pooled placebo | 2 | 0 (0.0) | 0 (0.0%) | 0 (0.0%) | 0 (0.0%) | 0 (0.0%) |
|  | D8 | Oxfendazole Cohort 1 | - | - | - | - | - | - |
|  |  | Oxfendazole Cohort 2 | - | - | - | - | - | - |
|  |  | Oxfendazole Cohort 3 | 8 | 0 (0.0) | 0 (0.0%) | 0 (0.0%) | 0 (0.0%) | 0 (0.0%) |
|  |  | Pooled placebo | 2 | 0 (0.0) | 0 (0.0%) | 0 (0.0%) | 0 (0.0%) | 0 (0.0%) |
|  | D14 | Oxfendazole Cohort 1 | 8 | 0 (0.0) | 1 (12.5%) | 0 (0.0%) | 0 (0.0%) | 0 (0.0%) |
|  |  | Oxfendazole Cohort 2 | 8 | 0 (0.0) | 2 (25.0%) | 0 (0.0%) | 0 (0.0%) | 0 (0.0%) |
|  |  | Oxfendazole Cohort 3 | 8 | 0 (0.0) | 3 (37.5%) | 0 (0.0%) | 0 (0.0%) | 0 (0.0%) |
|  |  | Pooled placebo | 6 | 0 (0.0) | 1 (16.7%) | 0 (0.0%) | 0 (0.0%) | 0 (0.0%) |

| Laboratory test (units) | Visit | Treatment  received |  |  |  | Result |  |  |
| --- | --- | --- | --- | --- | --- | --- | --- | --- |
|  |  |  | n expected | Missing, n (%) | Below reference but not clinically significant | Below reference and clinically significant | Above reference but not clinically significant | Above reference and clinically significant |
| Creatinine (µmol/L) | Baseline | Oxfendazole Cohort 1 | 8 | 0 (0.0) | 0 (0.0%) | 0 (0.0%) | 0 (0.0%) | 0 (0.0%) |
|  |  | Oxfendazole Cohort 2 | 8 | 0 (0.0) | 1 (12.5%) | 0 (0.0%) | 0 (0.0%) | 0 (0.0%) |
|  |  | Oxfendazole Cohort 3 | 8 | 0 (0.0) | 2 (25.0%) | 0 (0.0%) | 0 (0.0%) | 0 (0.0%) |
|  |  | Pooled placebo | 6 | 0 (0.0) | 2 (33.3%) | 0 (0.0%) | 0 (0.0%) | 0 (0.0%) |
|  | D2 | Oxfendazole Cohort 1 | 8 | 0 (0.0) | 1 (12.5%) | 0 (0.0%) | 0 (0.0%) | 0 (0.0%) |
|  |  | Oxfendazole Cohort 2 | 8 | 0 (0.0) | 1 (12.5%) | 0 (0.0%) | 1 (12.5%) | 0 (0.0%) |
|  |  | Oxfendazole Cohort 3 | 8 | 0 (0.0) | 1 (12.5%) | 0 (0.0%) | 0 (0.0%) | 0 (0.0%) |
|  |  | Pooled placebo | 6 | 0 (0.0) | 2 (33.3%) | 0 (0.0%) | 0 (0.0%) | 0 (0.0%) |
|  | D3 | Oxfendazole Cohort 1 | 8 | 0 (0.0) | 0 (0.0%) | 0 (0.0%) | 0 (0.0%) | 0 (0.0%) |
|  |  | Oxfendazole Cohort 2 | 8 | 0 (0.0) | 1 (12.5%) | 0 (0.0%) | 1 (12.5%) | 0 (0.0%) |
|  |  | Oxfendazole Cohort 3 | 8 | 0 (0.0) | 2 (25.0%) | 0 (0.0%) | 0 (0.0%) | 0 (0.0%) |
|  |  | Pooled placebo | 6 | 0 (0.0) | 2 (33.3%) | 0 (0.0%) | 0 (0.0%) | 0 (0.0%) |

| Laboratory test (units) | Visit | Treatment  received |  |  |  | Result |  |  |
| --- | --- | --- | --- | --- | --- | --- | --- | --- |
|  |  |  | n expected | Missing, n (%) | Below reference but not clinically significant | Below reference and clinically significant | Above reference but not clinically significant | Above reference and clinically significant |
|  | D5 | Oxfendazole Cohort 1 | - | - | - | - | - | - |
|  |  | Oxfendazole Cohort 2 | - | - | - | - | - | - |
|  |  | Oxfendazole Cohort 3 | 8 | 0 (0.0) | 2 (25.0%) | 0 (0.0%) | 0 (0.0%) | 0 (0.0%) |
|  |  | Pooled placebo | 2 | 0 (0.0) | 0 (0.0%) | 0 (0.0%) | 0 (0.0%) | 0 (0.0%) |
|  | D8 | Oxfendazole Cohort 1 | - | - | - | - | - | - |
|  |  | Oxfendazole Cohort 2 | - | - | - | - | - | - |
|  |  | Oxfendazole Cohort 3 | 8 | 0 (0.0) | 2 (25.0%) | 0 (0.0%) | 0 (0.0%) | 0 (0.0%) |
|  |  | Pooled placebo | 2 | 0 (0.0) | 0 (0.0%) | 0 (0.0%) | 0 (0.0%) | 0 (0.0%) |
|  | D14 | Oxfendazole Cohort 1 | 8 | 0 (0.0) | 0 (0.0%) | 0 (0.0%) | 0 (0.0%) | 0 (0.0%) |
|  |  | Oxfendazole Cohort 2 | 8 | 0 (0.0) | 1 (12.5%) | 0 (0.0%) | 1 (12.5%) | 0 (0.0%) |
|  |  | Oxfendazole Cohort 3 | 8 | 0 (0.0) | 3 (37.5%) | 0 (0.0%) | 0 (0.0%) | 0 (0.0%) |
|  |  | Pooled placebo | 6 | 0 (0.0) | 0 (0.0%) | 0 (0.0%) | 0 (0.0%) | 0 (0.0%) |

| Laboratory test (units) | Visit | Treatment  received |  |  |  | Result |  |  |
| --- | --- | --- | --- | --- | --- | --- | --- | --- |
|  |  |  | n expected | Missing, n (%) | Below reference but not clinically significant | Below reference and clinically significant | Above reference but not clinically significant | Above reference and clinically significant |
| Chloride (mmol/L) | Baseline | Oxfendazole Cohort 1 | 8 | 0 (0.0) | 1 (12.5%) | 0 (0.0%) | 0 (0.0%) | 0 (0.0%) |
|  |  | Oxfendazole Cohort 2 | 8 | 0 (0.0) | 0 (0.0%) | 0 (0.0%) | 0 (0.0%) | 0 (0.0%) |
|  |  | Oxfendazole Cohort 3 | 8 | 0 (0.0) | 0 (0.0%) | 0 (0.0%) | 1 (12.5%) | 0 (0.0%) |
|  |  | Pooled placebo | 6 | 0 (0.0) | 1 (16.7%) | 0 (0.0%) | 0 (0.0%) | 0 (0.0%) |
|  | D2 | Oxfendazole Cohort 1 | 8 | 0 (0.0) | 0 (0.0%) | 0 (0.0%) | 1 (12.5%) | 0 (0.0%) |
|  |  | Oxfendazole Cohort 2 | 8 | 0 (0.0) | 0 (0.0%) | 0 (0.0%) | 0 (0.0%) | 0 (0.0%) |
|  |  | Oxfendazole Cohort 3 | 8 | 0 (0.0) | 0 (0.0%) | 0 (0.0%) | 0 (0.0%) | 0 (0.0%) |
|  |  | Pooled placebo | 6 | 0 (0.0) | 1 (16.7%) | 0 (0.0%) | 0 (0.0%) | 0 (0.0%) |
|  | D3 | Oxfendazole Cohort 1 | 8 | 0 (0.0) | 0 (0.0%) | 0 (0.0%) | 1 (12.5%) | 0 (0.0%) |
|  |  | Oxfendazole Cohort 2 | 8 | 0 (0.0) | 0 (0.0%) | 0 (0.0%) | 0 (0.0%) | 0 (0.0%) |
|  |  | Oxfendazole Cohort 3 | 8 | 0 (0.0) | 0 (0.0%) | 0 (0.0%) | 0 (0.0%) | 0 (0.0%) |
|  |  | Pooled placebo | 6 | 0 (0.0) | 0 (0.0%) | 0 (0.0%) | 1 (16.7%) | 0 (0.0%) |

| Laboratory test (units) | Visit | Treatment  received |  |  |  | Result |  |  |
| --- | --- | --- | --- | --- | --- | --- | --- | --- |
|  |  |  | n expected | Missing, n (%) | Below reference but not clinically significant | Below reference and clinically significant | Above reference but not clinically significant | Above reference and clinically significant |
|  | D5 | Oxfendazole Cohort 1 | - | - | - | - | - | - |
|  |  | Oxfendazole Cohort 2 | - | - | - | - | - | - |
|  |  | Oxfendazole Cohort 3 | 8 | 0 (0.0) | 0 (0.0%) | 0 (0.0%) | 0 (0.0%) | 0 (0.0%) |
|  |  | Pooled placebo | 2 | 0 (0.0) | 0 (0.0%) | 0 (0.0%) | 0 (0.0%) | 0 (0.0%) |
|  | D8 | Oxfendazole Cohort 1 | - | - | - | - | - | - |
|  |  | Oxfendazole Cohort 2 | - | - | - | - | - | - |
|  |  | Oxfendazole Cohort 3 | 8 | 0 (0.0) | 0 (0.0%) | 0 (0.0%) | 0 (0.0%) | 0 (0.0%) |
|  |  | Pooled placebo | 2 | 0 (0.0) | 1 (50.0%) | 0 (0.0%) | 0 (0.0%) | 0 (0.0%) |
|  | D14 | Oxfendazole Cohort 1 | 8 | 0 (0.0) | 2 (25.0%) | 0 (0.0%) | 0 (0.0%) | 0 (0.0%) |
|  |  | Oxfendazole Cohort 2 | 8 | 0 (0.0) | 0 (0.0%) | 0 (0.0%) | 0 (0.0%) | 0 (0.0%) |
|  |  | Oxfendazole Cohort 3 | 8 | 0 (0.0) | 0 (0.0%) | 0 (0.0%) | 0 (0.0%) | 0 (0.0%) |
|  |  | Pooled placebo | 6 | 0 (0.0) | 0 (0.0%) | 0 (0.0%) | 0 (0.0%) | 0 (0.0%) |

| Laboratory test (units) | Visit | Treatment  received |  |  |  | Result |  |  |
| --- | --- | --- | --- | --- | --- | --- | --- | --- |
|  |  |  | n expected | Missing, n (%) | Below reference but not clinically significant | Below reference and clinically significant | Above reference but not clinically significant | Above reference and clinically significant |
| Bicarbonate (mmol/L) | Baseline | Oxfendazole Cohort 1 | 8 | 0 (0.0) | 4 (50.0%) | 0 (0.0%) | 0 (0.0%) | 0 (0.0%) |
|  |  | Oxfendazole Cohort 2 | 8 | 0 (0.0) | 5 (62.5%) | 0 (0.0%) | 0 (0.0%) | 0 (0.0%) |
|  |  | Oxfendazole Cohort 3 | 8 | 0 (0.0) | 1 (12.5%) | 0 (0.0%) | 0 (0.0%) | 0 (0.0%) |
|  |  | Pooled placebo | 6 | 0 (0.0) | 3 (50.0%) | 0 (0.0%) | 0 (0.0%) | 0 (0.0%) |
|  | D2 | Oxfendazole Cohort 1 | 8 | 0 (0.0) | 5 (62.5%) | 0 (0.0%) | 0 (0.0%) | 0 (0.0%) |
|  |  | Oxfendazole Cohort 2 | 8 | 0 (0.0) | 5 (62.5%) | 0 (0.0%) | 0 (0.0%) | 0 (0.0%) |
|  |  | Oxfendazole Cohort 3 | 8 | 0 (0.0) | 3 (37.5%) | 0 (0.0%) | 0 (0.0%) | 0 (0.0%) |
|  |  | Pooled placebo | 6 | 0 (0.0) | 5 (83.3%) | 0 (0.0%) | 0 (0.0%) | 0 (0.0%) |
|  | D3 | Oxfendazole Cohort 1 | 8 | 0 (0.0) | 5 (62.5%) | 0 (0.0%) | 0 (0.0%) | 0 (0.0%) |
|  |  | Oxfendazole Cohort 2 | 8 | 0 (0.0) | 5 (62.5%) | 0 (0.0%) | 0 (0.0%) | 0 (0.0%) |
|  |  | Oxfendazole Cohort 3 | 8 | 0 (0.0) | 2 (25.0%) | 0 (0.0%) | 0 (0.0%) | 0 (0.0%) |
|  |  | Pooled placebo | 6 | 0 (0.0) | 3 (50.0%) | 0 (0.0%) | 0 (0.0%) | 0 (0.0%) |

| Laboratory test (units) | Visit | Treatment  received |  |  |  | Result |  |  |
| --- | --- | --- | --- | --- | --- | --- | --- | --- |
|  |  |  | n expected | Missing, n (%) | Below reference but not clinically significant | Below reference and clinically significant | Above reference but not clinically significant | Above reference and clinically significant |
|  | D5 | Oxfendazole Cohort 1 | - | - | - | - | - | - |
|  |  | Oxfendazole Cohort 2 | - | - | - | - | - | - |
|  |  | Oxfendazole Cohort 3 | 8 | 0 (0.0) | 2 (25.0%) | 0 (0.0%) | 0 (0.0%) | 0 (0.0%) |
|  |  | Pooled placebo | 2 | 0 (0.0) | 2 (100.0%) | 0 (0.0%) | 0 (0.0%) | 0 (0.0%) |
|  | D8 | Oxfendazole Cohort 1 | - | - | - | - | - | - |
|  |  | Oxfendazole Cohort 2 | - | - | - | - | - | - |
|  |  | Oxfendazole Cohort 3 | 8 | 0 (0.0) | 1 (12.5%) | 0 (0.0%) | 0 (0.0%) | 0 (0.0%) |
|  |  | Pooled placebo | 2 | 0 (0.0) | 2 (100.0%) | 0 (0.0%) | 0 (0.0%) | 0 (0.0%) |
|  | D14 | Oxfendazole Cohort 1 | 8 | 0 (0.0) | 4 (50.0%) | 0 (0.0%) | 0 (0.0%) | 0 (0.0%) |
|  |  | Oxfendazole Cohort 2 | 8 | 0 (0.0) | 5 (62.5%) | 0 (0.0%) | 0 (0.0%) | 0 (0.0%) |
|  |  | Oxfendazole Cohort 3 | 8 | 0 (0.0) | 4 (50.0%) | 0 (0.0%) | 0 (0.0%) | 0 (0.0%) |
|  |  | Pooled placebo | 6 | 0 (0.0) | 4 (66.7%) | 0 (0.0%) | 0 (0.0%) | 0 (0.0%) |

| Laboratory test (units) | Visit | Treatment  received |  |  |  | Result |  |  |
| --- | --- | --- | --- | --- | --- | --- | --- | --- |
|  |  |  | n expected | Missing, n (%) | Below reference but not clinically significant | Below reference and clinically significant | Above reference but not clinically significant | Above reference and clinically significant |
| ALT (U/L) | Baseline | Oxfendazole Cohort 1 | 8 | 0 (0.0) | 0 (0.0%) | 0 (0.0%) | 0 (0.0%) | 0 (0.0%) |
|  |  | Oxfendazole Cohort 2 | 8 | 0 (0.0) | 0 (0.0%) | 0 (0.0%) | 0 (0.0%) | 0 (0.0%) |
|  |  | Oxfendazole Cohort 3 | 8 | 0 (0.0) | 0 (0.0%) | 0 (0.0%) | 1 (12.5%) | 0 (0.0%) |
|  |  | Pooled placebo | 6 | 0 (0.0) | 0 (0.0%) | 0 (0.0%) | 1 (16.7%) | 0 (0.0%) |
|  | D2 | Oxfendazole Cohort 1 | 8 | 0 (0.0) | 0 (0.0%) | 0 (0.0%) | 0 (0.0%) | 0 (0.0%) |
|  |  | Oxfendazole Cohort 2 | 8 | 0 (0.0) | 0 (0.0%) | 0 (0.0%) | 0 (0.0%) | 0 (0.0%) |
|  |  | Oxfendazole Cohort 3 | 8 | 0 (0.0) | 0 (0.0%) | 0 (0.0%) | 0 (0.0%) | 0 (0.0%) |
|  |  | Pooled placebo | 6 | 0 (0.0) | 0 (0.0%) | 0 (0.0%) | 1 (16.7%) | 0 (0.0%) |
|  | D3 | Oxfendazole Cohort 1 | 8 | 0 (0.0) | 0 (0.0%) | 0 (0.0%) | 0 (0.0%) | 0 (0.0%) |
|  |  | Oxfendazole Cohort 2 | 8 | 0 (0.0) | 0 (0.0%) | 0 (0.0%) | 0 (0.0%) | 0 (0.0%) |
|  |  | Oxfendazole Cohort 3 | 8 | 0 (0.0) | 0 (0.0%) | 0 (0.0%) | 0 (0.0%) | 0 (0.0%) |
|  |  | Pooled placebo | 6 | 0 (0.0) | 0 (0.0%) | 0 (0.0%) | 1 (16.7%) | 0 (0.0%) |

| Laboratory test (units) | Visit | Treatment  received |  |  |  | Result |  |  |
| --- | --- | --- | --- | --- | --- | --- | --- | --- |
|  |  |  | n expected | Missing, n (%) | Below reference but not clinically significant | Below reference and clinically significant | Above reference but not clinically significant | Above reference and clinically significant |
|  | D5 | Oxfendazole Cohort 1 | - | - | - | - | - | - |
|  |  | Oxfendazole Cohort 2 | - | - | - | - | - | - |
|  |  | Oxfendazole Cohort 3 | 8 | 0 (0.0) | 0 (0.0%) | 0 (0.0%) | 0 (0.0%) | 0 (0.0%) |
|  |  | Pooled placebo | 2 | 0 (0.0) | 0 (0.0%) | 0 (0.0%) | 0 (0.0%) | 0 (0.0%) |
|  | D8 | Oxfendazole Cohort 1 | - | - | - | - | - | - |
|  |  | Oxfendazole Cohort 2 | - | - | - | - | - | - |
|  |  | Oxfendazole Cohort 3 | 8 | 0 (0.0) | 0 (0.0%) | 0 (0.0%) | 0 (0.0%) | 0 (0.0%) |
|  |  | Pooled placebo | 2 | 0 (0.0) | 0 (0.0%) | 0 (0.0%) | 0 (0.0%) | 0 (0.0%) |
|  | D14 | Oxfendazole Cohort 1 | 8 | 0 (0.0) | 0 (0.0%) | 0 (0.0%) | 0 (0.0%) | 0 (0.0%) |
|  |  | Oxfendazole Cohort 2 | 8 | 0 (0.0) | 0 (0.0%) | 0 (0.0%) | 0 (0.0%) | 0 (0.0%) |
|  |  | Oxfendazole Cohort 3 | 8 | 0 (0.0) | 0 (0.0%) | 0 (0.0%) | 0 (0.0%) | 0 (0.0%) |
|  |  | Pooled placebo | 6 | 0 (0.0) | 0 (0.0%) | 0 (0.0%) | 0 (0.0%) | 0 (0.0%) |

| Laboratory test (units) | Visit | Treatment  received |  |  |  | Result |  |  |
| --- | --- | --- | --- | --- | --- | --- | --- | --- |
|  |  |  | n expected | Missing, n (%) | Below reference but not clinically significant | Below reference and clinically significant | Above reference but not clinically significant | Above reference and clinically significant |
| AST (U/L) | Baseline | Oxfendazole Cohort 1 | 8 | 0 (0.0) | 0 (0.0%) | 0 (0.0%) | 0 (0.0%) | 0 (0.0%) |
|  |  | Oxfendazole Cohort 2 | 8 | 0 (0.0) | 0 (0.0%) | 0 (0.0%) | 0 (0.0%) | 0 (0.0%) |
|  |  | Oxfendazole Cohort 3 | 8 | 0 (0.0) | 3 (37.5%) | 0 (0.0%) | 0 (0.0%) | 0 (0.0%) |
|  |  | Pooled placebo | 6 | 0 (0.0) | 0 (0.0%) | 0 (0.0%) | 0 (0.0%) | 0 (0.0%) |
|  | D2 | Oxfendazole Cohort 1 | 8 | 0 (0.0) | 0 (0.0%) | 0 (0.0%) | 0 (0.0%) | 0 (0.0%) |
|  |  | Oxfendazole Cohort 2 | 8 | 0 (0.0) | 0 (0.0%) | 0 (0.0%) | 0 (0.0%) | 0 (0.0%) |
|  |  | Oxfendazole Cohort 3 | 8 | 0 (0.0) | 2 (25.0%) | 0 (0.0%) | 0 (0.0%) | 0 (0.0%) |
|  |  | Pooled placebo | 6 | 0 (0.0) | 1 (16.7%) | 0 (0.0%) | 0 (0.0%) | 0 (0.0%) |
|  | D3 | Oxfendazole Cohort 1 | 8 | 0 (0.0) | 0 (0.0%) | 0 (0.0%) | 0 (0.0%) | 0 (0.0%) |
|  |  | Oxfendazole Cohort 2 | 8 | 0 (0.0) | 0 (0.0%) | 0 (0.0%) | 0 (0.0%) | 0 (0.0%) |
|  |  | Oxfendazole Cohort 3 | 8 | 0 (0.0) | 2 (25.0%) | 0 (0.0%) | 0 (0.0%) | 0 (0.0%) |
|  |  | Pooled placebo | 6 | 0 (0.0) | 0 (0.0%) | 0 (0.0%) | 0 (0.0%) | 0 (0.0%) |

| Laboratory test (units) | Visit | Treatment  received |  |  |  | Result |  |  |
| --- | --- | --- | --- | --- | --- | --- | --- | --- |
|  |  |  | n expected | Missing, n (%) | Below reference but not clinically significant | Below reference and clinically significant | Above reference but not clinically significant | Above reference and clinically significant |
|  | D5 | Oxfendazole Cohort 1 | - | - | - | - | - | - |
|  |  | Oxfendazole Cohort 2 | - | - | - | - | - | - |
|  |  | Oxfendazole Cohort 3 | 8 | 0 (0.0) | 1 (12.5%) | 0 (0.0%) | 0 (0.0%) | 0 (0.0%) |
|  |  | Pooled placebo | 2 | 0 (0.0) | 0 (0.0%) | 0 (0.0%) | 0 (0.0%) | 0 (0.0%) |
|  | D8 | Oxfendazole Cohort 1 | - | - | - | - | - | - |
|  |  | Oxfendazole Cohort 2 | - | - | - | - | - | - |
|  |  | Oxfendazole Cohort 3 | 8 | 0 (0.0) | 0 (0.0%) | 0 (0.0%) | 0 (0.0%) | 0 (0.0%) |
|  |  | Pooled placebo | 2 | 0 (0.0) | 0 (0.0%) | 0 (0.0%) | 0 (0.0%) | 0 (0.0%) |
|  | D14 | Oxfendazole Cohort 1 | 8 | 0 (0.0) | 0 (0.0%) | 0 (0.0%) | 0 (0.0%) | 0 (0.0%) |
|  |  | Oxfendazole Cohort 2 | 8 | 0 (0.0) | 0 (0.0%) | 0 (0.0%) | 0 (0.0%) | 0 (0.0%) |
|  |  | Oxfendazole Cohort 3 | 8 | 0 (0.0) | 2 (25.0%) | 0 (0.0%) | 0 (0.0%) | 0 (0.0%) |
|  |  | Pooled placebo | 6 | 0 (0.0) | 0 (0.0%) | 0 (0.0%) | 0 (0.0%) | 0 (0.0%) |

| Laboratory test (units) | Visit | Treatment  received |  |  |  | Result |  |  |
| --- | --- | --- | --- | --- | --- | --- | --- | --- |
|  |  |  | n expected | Missing, n (%) | Below reference but not clinically significant | Below reference and clinically significant | Above reference but not clinically significant | Above reference and clinically significant |
| Bilirubin (µmol/L) | Baseline | Oxfendazole Cohort 1 | 8 | 0 (0.0) | 0 (0.0%) | 0 (0.0%) | 0 (0.0%) | 0 (0.0%) |
|  |  | Oxfendazole Cohort 2 | 8 | 0 (0.0) | 0 (0.0%) | 0 (0.0%) | 0 (0.0%) | 0 (0.0%) |
|  |  | Oxfendazole Cohort 3 | 8 | 0 (0.0) | 0 (0.0%) | 0 (0.0%) | 0 (0.0%) | 0 (0.0%) |
|  |  | Pooled placebo | 6 | 0 (0.0) | 0 (0.0%) | 0 (0.0%) | 0 (0.0%) | 0 (0.0%) |
|  | D2 | Oxfendazole Cohort 1 | 8 | 0 (0.0) | 0 (0.0%) | 0 (0.0%) | 0 (0.0%) | 0 (0.0%) |
|  |  | Oxfendazole Cohort 2 | 8 | 0 (0.0) | 0 (0.0%) | 0 (0.0%) | 0 (0.0%) | 0 (0.0%) |
|  |  | Oxfendazole Cohort 3 | 8 | 0 (0.0) | 1 (12.5%) | 0 (0.0%) | 0 (0.0%) | 0 (0.0%) |
|  |  | Pooled placebo | 6 | 0 (0.0) | 2 (33.3%) | 0 (0.0%) | 0 (0.0%) | 0 (0.0%) |
|  | D3 | Oxfendazole Cohort 1 | 8 | 0 (0.0) | 0 (0.0%) | 0 (0.0%) | 0 (0.0%) | 0 (0.0%) |
|  |  | Oxfendazole Cohort 2 | 8 | 0 (0.0) | 1 (12.5%) | 0 (0.0%) | 0 (0.0%) | 0 (0.0%) |
|  |  | Oxfendazole Cohort 3 | 8 | 0 (0.0) | 1 (12.5%) | 0 (0.0%) | 0 (0.0%) | 0 (0.0%) |
|  |  | Pooled placebo | 6 | 0 (0.0) | 1 (16.7%) | 0 (0.0%) | 0 (0.0%) | 0 (0.0%) |

| Laboratory test (units) | Visit | Treatment  received |  |  |  | Result |  |  |
| --- | --- | --- | --- | --- | --- | --- | --- | --- |
|  |  |  | n expected | Missing, n (%) | Below reference but not clinically significant | Below reference and clinically significant | Above reference but not clinically significant | Above reference and clinically significant |
|  | D5 | Oxfendazole Cohort 1 | - | - | - | - | - | - |
|  |  | Oxfendazole Cohort 2 | - | - | - | - | - | - |
|  |  | Oxfendazole Cohort 3 | 8 | 0 (0.0) | 1 (12.5%) | 0 (0.0%) | 0 (0.0%) | 0 (0.0%) |
|  |  | Pooled placebo | 2 | 0 (0.0) | 0 (0.0%) | 0 (0.0%) | 0 (0.0%) | 0 (0.0%) |
|  | D8 | Oxfendazole Cohort 1 | - | - | - | - | - | - |
|  |  | Oxfendazole Cohort 2 | - | - | - | - | - | - |
|  |  | Oxfendazole Cohort 3 | 8 | 0 (0.0) | 2 (25.0%) | 0 (0.0%) | 0 (0.0%) | 0 (0.0%) |
|  |  | Pooled placebo | 2 | 0 (0.0) | 0 (0.0%) | 0 (0.0%) | 0 (0.0%) | 0 (0.0%) |
|  | D14 | Oxfendazole Cohort 1 | 8 | 0 (0.0) | 0 (0.0%) | 0 (0.0%) | 0 (0.0%) | 0 (0.0%) |
|  |  | Oxfendazole Cohort 2 | 8 | 0 (0.0) | 0 (0.0%) | 0 (0.0%) | 0 (0.0%) | 0 (0.0%) |
|  |  | Oxfendazole Cohort 3 | 8 | 0 (0.0) | 0 (0.0%) | 0 (0.0%) | 0 (0.0%) | 0 (0.0%) |
|  |  | Pooled placebo | 6 | 0 (0.0) | 0 (0.0%) | 0 (0.0%) | 0 (0.0%) | 0 (0.0%) |

Based on safety set.

Note: Participants who have been withdrawn from study or not attended a visit are not counted in the expected number. Only two placebo participants from cohort 3 are expected at Day3 - Day8.

Please note that listings below only include abnormal biochemistry results recorded after baseline.

S10 Summary of non-clinically significant, abnormal hematology results

| Laboratory test (units) | Visit | Treatment received | Result | | | | | |
| --- | --- | --- | --- | --- | --- | --- | --- | --- |
|  |  |  | n expected | Missing, n (%) | Below reference but not clinically significant | Below reference and clinically significant | Above reference but not clinically significant | Above reference and clinically significant |
| Haemoglobin (g/dL) | Baseline | Oxfendazole Cohort 1 | 8 | 0 (0.0) | 1 (12.5%) | 0 (0.0%) | 0 (0.0%) | 0 (0.0%) |
|  |  | Oxfendazole Cohort 2 | 8 | 0 (0.0) | 0 (0.0%) | 0 (0.0%) | 0 (0.0%) | 0 (0.0%) |
|  |  | Oxfendazole Cohort 3 | 8 | 0 (0.0) | 0 (0.0%) | 0 (0.0%) | 0 (0.0%) | 0 (0.0%) |
|  |  | Pooled placebo | 6 | 0 (0.0) | 0 (0.0%) | 0 (0.0%) | 0 (0.0%) | 0 (0.0%) |
|  | D2 | Oxfendazole Cohort 1 | 8 | 0 (0.0) | 1 (12.5%) | 0 (0.0%) | 0 (0.0%) | 0 (0.0%) |
|  |  | Oxfendazole Cohort 2 | 8 | 0 (0.0) | 0 (0.0%) | 0 (0.0%) | 0 (0.0%) | 0 (0.0%) |
|  |  | Oxfendazole Cohort 3 | 8 | 0 (0.0) | 0 (0.0%) | 0 (0.0%) | 0 (0.0%) | 0 (0.0%) |
|  |  | Pooled placebo | 6 | 0 (0.0) | 0 (0.0%) | 0 (0.0%) | 0 (0.0%) | 0 (0.0%) |
|  | D3 | Oxfendazole Cohort 1 | 8 | 0 (0.0) | 0 (0.0%) | 0 (0.0%) | 0 (0.0%) | 0 (0.0%) |
|  |  | Oxfendazole Cohort 2 | 8 | 0 (0.0) | 0 (0.0%) | 0 (0.0%) | 0 (0.0%) | 0 (0.0%) |
|  |  | Oxfendazole Cohort 3 | 8 | 0 (0.0) | 0 (0.0%) | 0 (0.0%) | 0 (0.0%) | 0 (0.0%) |
|  |  | Pooled placebo | 6 | 0 (0.0) | 0 (0.0%) | 0 (0.0%) | 0 (0.0%) | 0 (0.0%) |
|  | D5 | Oxfendazole Cohort 1 | - | - | - | - | - | - |
|  |  | Oxfendazole Cohort 2 | - | - | - | - | - | - |
|  |  | Oxfendazole Cohort 3 | 8 | 0 (0.0) | 0 (0.0%) | 0 (0.0%) | 0 (0.0%) | 0 (0.0%) |
|  |  | Pooled placebo | 2 | 0 (0.0) | 0 (0.0%) | 0 (0.0%) | 0 (0.0%) | 0 (0.0%) |
|  | D8 | Oxfendazole Cohort 1 | - | - | - | - | - | - |
|  |  | Oxfendazole Cohort 2 | - | - | - | - | - | - |
|  |  | Oxfendazole Cohort 3 | 8 | 0 (0.0) | 0 (0.0%) | 0 (0.0%) | 0 (0.0%) | 0 (0.0%) |
|  |  | Pooled placebo | 2 | 0 (0.0) | 0 (0.0%) | 0 (0.0%) | 0 (0.0%) | 0 (0.0%) |
|  | D14 | Oxfendazole Cohort 1 | 8 | 0 (0.0) | 1 (12.5%) | 0 (0.0%) | 0 (0.0%) | 0 (0.0%) |
|  |  | Oxfendazole Cohort 2 | 8 | 0 (0.0) | 0 (0.0%) | 0 (0.0%) | 0 (0.0%) | 0 (0.0%) |
|  |  | Oxfendazole Cohort 3 | 8 | 0 (0.0) | 0 (0.0%) | 0 (0.0%) | 0 (0.0%) | 0 (0.0%) |
|  |  | Pooled placebo | 6 | 0 (0.0) | 0 (0.0%) | 0 (0.0%) | 0 (0.0%) | 0 (0.0%) |
| Leucocyte count WBC (10^3/µL) | Baseline | Oxfendazole Cohort 1 | 8 | 0 (0.0) | 0 (0.0%) | 0 (0.0%) | 0 (0.0%) | 0 (0.0%) |
|  |  | Oxfendazole Cohort 2 | 8 | 0 (0.0) | 2 (25.0%) | 0 (0.0%) | 0 (0.0%) | 0 (0.0%) |
|  |  | Oxfendazole Cohort 3 | 8 | 0 (0.0) | 1 (12.5%) | 0 (0.0%) | 0 (0.0%) | 0 (0.0%) |
|  |  | Pooled placebo | 6 | 0 (0.0) | 0 (0.0%) | 0 (0.0%) | 0 (0.0%) | 0 (0.0%) |
|  | D2 | Oxfendazole Cohort 1 | 8 | 0 (0.0) | 1 (12.5%) | 0 (0.0%) | 0 (0.0%) | 0 (0.0%) |
|  |  | Oxfendazole Cohort 2 | 8 | 0 (0.0) | 1 (12.5%) | 0 (0.0%) | 0 (0.0%) | 0 (0.0%) |
|  |  | Oxfendazole Cohort 3 | 8 | 0 (0.0) | 0 (0.0%) | 0 (0.0%) | 0 (0.0%) | 0 (0.0%) |
|  |  | Pooled placebo | 6 | 0 (0.0) | 1 (16.7%) | 0 (0.0%) | 0 (0.0%) | 0 (0.0%) |
|  | D3 | Oxfendazole Cohort 1 | 8 | 0 (0.0) | 1 (12.5%) | 0 (0.0%) | 0 (0.0%) | 0 (0.0%) |
|  |  | Oxfendazole Cohort 2 | 8 | 0 (0.0) | 0 (0.0%) | 0 (0.0%) | 0 (0.0%) | 0 (0.0%) |
|  |  | Oxfendazole Cohort 3 | 8 | 0 (0.0) | 1 (12.5%) | 0 (0.0%) | 0 (0.0%) | 0 (0.0%) |
|  |  | Pooled placebo | 6 | 0 (0.0) | 0 (0.0%) | 0 (0.0%) | 0 (0.0%) | 0 (0.0%) |
|  | D5 | Oxfendazole Cohort 1 | - | - | - | - | - | - |
|  |  | Oxfendazole Cohort 2 | - | - | - | - | - | - |
|  |  | Oxfendazole Cohort 3 | 8 | 0 (0.0) | 1 (12.5%) | 0 (0.0%) | 1 (12.5%) | 0 (0.0%) |
|  |  | Pooled placebo | 2 | 0 (0.0) | 0 (0.0%) | 0 (0.0%) | 0 (0.0%) | 0 (0.0%) |
|  | D8 | Oxfendazole Cohort 1 | - | - | - | - | - | - |
|  |  | Oxfendazole Cohort 2 | - | - | - | - | - | - |
|  |  | Oxfendazole Cohort 3 | 8 | 0 (0.0) | 0 (0.0%) | 0 (0.0%) | 1 (12.5%) | 0 (0.0%) |
|  |  | Pooled placebo | 2 | 0 (0.0) | 0 (0.0%) | 0 (0.0%) | 0 (0.0%) | 0 (0.0%) |
|  | D14 | Oxfendazole Cohort 1 | 8 | 0 (0.0) | 0 (0.0%) | 0 (0.0%) | 0 (0.0%) | 0 (0.0%) |
|  |  | Oxfendazole Cohort 2 | 8 | 0 (0.0) | 1 (12.5%) | 0 (0.0%) | 0 (0.0%) | 0 (0.0%) |
|  |  | Oxfendazole Cohort 3 | 8 | 0 (0.0) | 0 (0.0%) | 0 (0.0%) | 0 (0.0%) | 0 (0.0%) |
|  |  | Pooled placebo | 6 | 0 (0.0) | 1 (16.7%) | 0 (0.0%) | 0 (0.0%) | 0 (0.0%) |
| Neutrophils (10^3/µL) | Baseline | Oxfendazole Cohort 1 | 8 | 0 (0.0) | 0 (0.0%) | 0 (0.0%) | 0 (0.0%) | 0 (0.0%) |
|  |  | Oxfendazole Cohort 2 | 8 | 0 (0.0) | 0 (0.0%) | 0 (0.0%) | 0 (0.0%) | 0 (0.0%) |
|  |  | Oxfendazole Cohort 3 | 8 | 0 (0.0) | 1 (12.5%) | 0 (0.0%) | 1 (12.5%) | 0 (0.0%) |
|  |  | Pooled placebo | 6 | 0 (0.0) | 0 (0.0%) | 0 (0.0%) | 0 (0.0%) | 0 (0.0%) |
|  | D2 | Oxfendazole Cohort 1 | 8 | 0 (0.0) | 1 (12.5%) | 0 (0.0%) | 0 (0.0%) | 0 (0.0%) |
|  |  | Oxfendazole Cohort 2 | 8 | 0 (0.0) | 1 (12.5%) | 0 (0.0%) | 0 (0.0%) | 0 (0.0%) |
|  |  | Oxfendazole Cohort 3 | 8 | 0 (0.0) | 0 (0.0%) | 0 (0.0%) | 0 (0.0%) | 0 (0.0%) |
|  |  | Pooled placebo | 6 | 0 (0.0) | 0 (0.0%) | 0 (0.0%) | 0 (0.0%) | 0 (0.0%) |
|  | D3 | Oxfendazole Cohort 1 | 8 | 0 (0.0) | 1 (12.5%) | 0 (0.0%) | 0 (0.0%) | 0 (0.0%) |
|  |  | Oxfendazole Cohort 2 | 8 | 0 (0.0) | 1 (12.5%) | 0 (0.0%) | 0 (0.0%) | 0 (0.0%) |
|  |  | Oxfendazole Cohort 3 | 8 | 0 (0.0) | 1 (12.5%) | 0 (0.0%) | 0 (0.0%) | 0 (0.0%) |
|  |  | Pooled placebo | 6 | 0 (0.0) | 0 (0.0%) | 0 (0.0%) | 0 (0.0%) | 0 (0.0%) |
|  | D5 | Oxfendazole Cohort 1 | - | - | - | - | - | - |
|  |  | Oxfendazole Cohort 2 | - | - | - | - | - | - |
|  |  | Oxfendazole Cohort 3 | 8 | 0 (0.0) | 1 (12.5%) | 0 (0.0%) | 0 (0.0%) | 0 (0.0%) |
|  |  | Pooled placebo | 2 | 0 (0.0) | 0 (0.0%) | 0 (0.0%) | 0 (0.0%) | 0 (0.0%) |
|  | D8 | Oxfendazole Cohort 1 | - | - | - | - | - | - |
|  |  | Oxfendazole Cohort 2 | - | - | - | - | - | - |
|  |  | Oxfendazole Cohort 3 | 8 | 0 (0.0) | 0 (0.0%) | 0 (0.0%) | 1 (12.5%) | 0 (0.0%) |
|  |  | Pooled placebo | 2 | 0 (0.0) | 0 (0.0%) | 0 (0.0%) | 0 (0.0%) | 0 (0.0%) |
|  | D14 | Oxfendazole Cohort 1 | 8 | 0 (0.0) | 0 (0.0%) | 0 (0.0%) | 0 (0.0%) | 0 (0.0%) |
|  |  | Oxfendazole Cohort 2 | 8 | 0 (0.0) | 0 (0.0%) | 0 (0.0%) | 0 (0.0%) | 0 (0.0%) |
|  |  | Oxfendazole Cohort 3 | 8 | 0 (0.0) | 1 (12.5%) | 0 (0.0%) | 0 (0.0%) | 0 (0.0%) |
|  |  | Pooled placebo | 6 | 0 (0.0) | 1 (16.7%) | 0 (0.0%) | 0 (0.0%) | 0 (0.0%) |
| Eosinophils (10^3/µL) | Baseline | Oxfendazole Cohort 1 | 8 | 0 (0.0) | 0 (0.0%) | 0 (0.0%) | 0 (0.0%) | 0 (0.0%) |
|  |  | Oxfendazole Cohort 2 | 8 | 0 (0.0) | 0 (0.0%) | 0 (0.0%) | 0 (0.0%) | 0 (0.0%) |
|  |  | Oxfendazole Cohort 3 | 8 | 0 (0.0) | 0 (0.0%) | 0 (0.0%) | 0 (0.0%) | 0 (0.0%) |
|  |  | Pooled placebo | 6 | 0 (0.0) | 0 (0.0%) | 0 (0.0%) | 0 (0.0%) | 0 (0.0%) |
|  | D2 | Oxfendazole Cohort 1 | 8 | 0 (0.0) | 0 (0.0%) | 0 (0.0%) | 0 (0.0%) | 0 (0.0%) |
|  |  | Oxfendazole Cohort 2 | 8 | 0 (0.0) | 0 (0.0%) | 0 (0.0%) | 0 (0.0%) | 0 (0.0%) |
|  |  | Oxfendazole Cohort 3 | 8 | 0 (0.0) | 0 (0.0%) | 0 (0.0%) | 0 (0.0%) | 0 (0.0%) |
|  |  | Pooled placebo | 6 | 0 (0.0) | 0 (0.0%) | 0 (0.0%) | 0 (0.0%) | 0 (0.0%) |
|  | D3 | Oxfendazole Cohort 1 | 8 | 0 (0.0) | 0 (0.0%) | 0 (0.0%) | 0 (0.0%) | 0 (0.0%) |
|  |  | Oxfendazole Cohort 2 | 8 | 0 (0.0) | 0 (0.0%) | 0 (0.0%) | 0 (0.0%) | 0 (0.0%) |
|  |  | Oxfendazole Cohort 3 | 8 | 0 (0.0) | 0 (0.0%) | 0 (0.0%) | 0 (0.0%) | 0 (0.0%) |
|  |  | Pooled placebo | 6 | 0 (0.0) | 0 (0.0%) | 0 (0.0%) | 0 (0.0%) | 0 (0.0%) |
|  | D5 | Oxfendazole Cohort 1 | - | - | - | - | - | - |
|  |  | Oxfendazole Cohort 2 | - | - | - | - | - | - |
|  |  | Oxfendazole Cohort 3 | 8 | 0 (0.0) | 0 (0.0%) | 0 (0.0%) | 0 (0.0%) | 0 (0.0%) |
|  |  | Pooled placebo | 2 | 0 (0.0) | 0 (0.0%) | 0 (0.0%) | 0 (0.0%) | 0 (0.0%) |
|  | D8 | Oxfendazole Cohort 1 | - | - | - | - | - | - |
|  |  | Oxfendazole Cohort 2 | - | - | - | - | - | - |
|  |  | Oxfendazole Cohort 3 | 8 | 0 (0.0) | 0 (0.0%) | 0 (0.0%) | 0 (0.0%) | 0 (0.0%) |
|  |  | Pooled placebo | 2 | 0 (0.0) | 0 (0.0%) | 0 (0.0%) | 0 (0.0%) | 0 (0.0%) |
|  | D14 | Oxfendazole Cohort 1 | 8 | 0 (0.0) | 0 (0.0%) | 0 (0.0%) | 0 (0.0%) | 0 (0.0%) |
|  |  | Oxfendazole Cohort 2 | 8 | 0 (0.0) | 0 (0.0%) | 0 (0.0%) | 0 (0.0%) | 0 (0.0%) |
|  |  | Oxfendazole Cohort 3 | 8 | 0 (0.0) | 0 (0.0%) | 0 (0.0%) | 0 (0.0%) | 0 (0.0%) |
|  |  | Pooled placebo | 6 | 0 (0.0) | 0 (0.0%) | 0 (0.0%) | 0 (0.0%) | 0 (0.0%) |
| Platelets (10^3/µL) | Baseline | Oxfendazole Cohort 1 | 8 | 0 (0.0) | 0 (0.0%) | 0 (0.0%) | 0 (0.0%) | 0 (0.0%) |
|  |  | Oxfendazole Cohort 2 | 8 | 0 (0.0) | 0 (0.0%) | 0 (0.0%) | 0 (0.0%) | 0 (0.0%) |
|  |  | Oxfendazole Cohort 3 | 8 | 0 (0.0) | 0 (0.0%) | 0 (0.0%) | 0 (0.0%) | 0 (0.0%) |
|  |  | Pooled placebo | 6 | 0 (0.0) | 0 (0.0%) | 0 (0.0%) | 0 (0.0%) | 0 (0.0%) |
|  | D2 | Oxfendazole Cohort 1 | 8 | 0 (0.0) | 0 (0.0%) | 0 (0.0%) | 0 (0.0%) | 0 (0.0%) |
|  |  | Oxfendazole Cohort 2 | 8 | 0 (0.0) | 1 (12.5%) | 0 (0.0%) | 0 (0.0%) | 0 (0.0%) |
|  |  | Oxfendazole Cohort 3 | 8 | 0 (0.0) | 0 (0.0%) | 0 (0.0%) | 0 (0.0%) | 0 (0.0%) |
|  |  | Pooled placebo | 6 | 0 (0.0) | 0 (0.0%) | 0 (0.0%) | 0 (0.0%) | 0 (0.0%) |
|  | D3 | Oxfendazole Cohort 1 | 8 | 0 (0.0) | 0 (0.0%) | 0 (0.0%) | 0 (0.0%) | 0 (0.0%) |
|  |  | Oxfendazole Cohort 2 | 8 | 0 (0.0) | 1 (12.5%) | 0 (0.0%) | 0 (0.0%) | 0 (0.0%) |
|  |  | Oxfendazole Cohort 3 | 8 | 0 (0.0) | 0 (0.0%) | 0 (0.0%) | 0 (0.0%) | 0 (0.0%) |
|  |  | Pooled placebo | 6 | 0 (0.0) | 0 (0.0%) | 0 (0.0%) | 0 (0.0%) | 0 (0.0%) |
|  | D5 | Oxfendazole Cohort 1 | - | - | - | - | - | - |
|  |  | Oxfendazole Cohort 2 | - | - | - | - | - | - |
|  |  | Oxfendazole Cohort 3 | 8 | 0 (0.0) | 0 (0.0%) | 0 (0.0%) | 0 (0.0%) | 0 (0.0%) |
|  |  | Pooled placebo | 2 | 0 (0.0) | 0 (0.0%) | 0 (0.0%) | 0 (0.0%) | 0 (0.0%) |
|  | D8 | Oxfendazole Cohort 1 | - | - | - | - | - | - |
|  |  | Oxfendazole Cohort 2 | - | - | - | - | - | - |
|  |  | Oxfendazole Cohort 3 | 8 | 0 (0.0) | 0 (0.0%) | 0 (0.0%) | 0 (0.0%) | 0 (0.0%) |
|  |  | Pooled placebo | 2 | 0 (0.0) | 0 (0.0%) | 0 (0.0%) | 0 (0.0%) | 0 (0.0%) |
|  | D14 | Oxfendazole Cohort 1 | 8 | 0 (0.0) | 0 (0.0%) | 0 (0.0%) | 0 (0.0%) | 0 (0.0%) |
|  |  | Oxfendazole Cohort 2 | 8 | 0 (0.0) | 1 (12.5%) | 0 (0.0%) | 2 (25.0%) | 0 (0.0%) |
|  |  | Oxfendazole Cohort 3 | 8 | 0 (0.0) | 0 (0.0%) | 0 (0.0%) | 0 (0.0%) | 0 (0.0%) |
|  |  | Pooled placebo | 6 | 0 (0.0) | 0 (0.0%) | 0 (0.0%) | 0 (0.0%) | 0 (0.0%) |

Based on safety set.

Note: Participants who have been withdrawn from study or not attended a visit are not counted in the expected number. Only two placebo participants from Cohort 3 are expected at Day 3 – Day 8.

Please note that listings below only include abnormal haematology results recorded after baseline.

S11 Summary of non-clinically significant, abnormal coagulation results

| Laboratory test (units) | Visit | Treatment received | Result | | | | | |
| --- | --- | --- | --- | --- | --- | --- | --- | --- |
|  |  |  | n expected | Missing, n (%) | Below reference but not clinically significant | Below reference and clinically significant | Above reference but not clinically significant | Above reference and clinically significant |
| Prothrombin time (sec) | Baseline | Oxfendazole Cohort 1 | 8 | 0 (0.0) | 1 (12.5%) | 0 (0.0%) | 1 (12.5%) | 0 (0.0%) |
|  |  | Oxfendazole Cohort 2 | 8 | 0 (0.0) | 0 (0.0%) | 0 (0.0%) | 0 (0.0%) | 0 (0.0%) |
|  |  | Oxfendazole Cohort 3 | 8 | 0 (0.0) | 2 (25.0%) | 0 (0.0%) | 0 (0.0%) | 0 (0.0%) |
|  |  | Pooled placebo | 6 | 0 (0.0) | 1 (16.7%) | 0 (0.0%) | 0 (0.0%) | 0 (0.0%) |
|  | D2 | Oxfendazole Cohort 1 | 8 | 0 (0.0) | 1 (12.5%) | 0 (0.0%) | 0 (0.0%) | 0 (0.0%) |
|  |  | Oxfendazole Cohort 2 | 8 | 0 (0.0) | 4 (50.0%) | 0 (0.0%) | 0 (0.0%) | 0 (0.0%) |
|  |  | Oxfendazole Cohort 3 | 8 | 0 (0.0) | 4 (50.0%) | 0 (0.0%) | 0 (0.0%) | 0 (0.0%) |
|  |  | Pooled placebo | 6 | 0 (0.0) | 2 (33.3%) | 0 (0.0%) | 0 (0.0%) | 0 (0.0%) |
|  | D3 | Oxfendazole Cohort 1 | 8 | 0 (0.0) | 1 (12.5%) | 0 (0.0%) | 0 (0.0%) | 0 (0.0%) |
|  |  | Oxfendazole Cohort 2 | 8 | 0 (0.0) | 5 (62.5%) | 0 (0.0%) | 1 (12.5%) | 0 (0.0%) |
|  |  | Oxfendazole Cohort 3 | 8 | 0 (0.0) | 4 (50.0%) | 0 (0.0%) | 0 (0.0%) | 0 (0.0%) |
|  |  | Pooled placebo | 6 | 0 (0.0) | 0 (0.0%) | 0 (0.0%) | 0 (0.0%) | 0 (0.0%) |
|  | D5 | Oxfendazole Cohort 1 | - | - | - | - | - | - |
|  |  | Oxfendazole Cohort 2 | - | - | - | - | - | - |
|  |  | Oxfendazole Cohort 3 | 8 | 0 (0.0) | 4 (50.0%) | 0 (0.0%) | 0 (0.0%) | 0 (0.0%) |
|  |  | Pooled placebo | 2 | 0 (0.0) | 0 (0.0%) | 0 (0.0%) | 0 (0.0%) | 0 (0.0%) |
|  | D8 | Oxfendazole Cohort 1 | - | - | - | - | - | - |
|  |  | Oxfendazole Cohort 2 | - | - | - | - | - | - |
|  |  | Oxfendazole Cohort 3 | 8 | 0 (0.0) | 5 (62.5%) | 0 (0.0%) | 0 (0.0%) | 0 (0.0%) |
|  |  | Pooled placebo | 2 | 0 (0.0) | 0 (0.0%) | 0 (0.0%) | 0 (0.0%) | 0 (0.0%) |
|  | D14 | Oxfendazole Cohort 1 | 8 | 0 (0.0) | 0 (0.0%) | 0 (0.0%) | 0 (0.0%) | 0 (0.0%) |
|  |  | Oxfendazole Cohort 2 | 8 | 0 (0.0) | 2 (25.0%) | 0 (0.0%) | 1 (12.5%) | 0 (0.0%) |
|  |  | Oxfendazole Cohort 3 | 8 | 0 (0.0) | 2 (25.0%) | 0 (0.0%) | 0 (0.0%) | 0 (0.0%) |
|  |  | Pooled placebo | 6 | 0 (0.0) | 1 (16.7%) | 0 (0.0%) | 0 (0.0%) | 0 (0.0%) |
| Activated partial thromboplastin time (sec) | Baseline | Oxfendazole Cohort 1 | 8 | 0 (0.0) | 0 (0.0%) | 0 (0.0%) | 4 (50.0%) | 0 (0.0%) |
|  |  | Oxfendazole Cohort 2 | 8 | 0 (0.0) | 0 (0.0%) | 0 (0.0%) | 3 (37.5%) | 0 (0.0%) |
|  |  | Oxfendazole Cohort 3 | 8 | 0 (0.0) | 0 (0.0%) | 0 (0.0%) | 1 (12.5%) | 0 (0.0%) |
|  |  | Pooled placebo | 6 | 0 (0.0) | 0 (0.0%) | 0 (0.0%) | 2 (33.3%) | 0 (0.0%) |
|  | D2 | Oxfendazole Cohort 1 | 8 | 0 (0.0) | 0 (0.0%) | 0 (0.0%) | 2 (25.0%) | 0 (0.0%) |
|  |  | Oxfendazole Cohort 2 | 8 | 0 (0.0) | 0 (0.0%) | 0 (0.0%) | 1 (12.5%) | 0 (0.0%) |
|  |  | Oxfendazole Cohort 3 | 8 | 0 (0.0) | 1 (12.5%) | 0 (0.0%) | 3 (37.5%) | 0 (0.0%) |
|  |  | Pooled placebo | 6 | 0 (0.0) | 0 (0.0%) | 0 (0.0%) | 2 (33.3%) | 0 (0.0%) |
|  | D3 | Oxfendazole Cohort 1 | 8 | 0 (0.0) | 0 (0.0%) | 0 (0.0%) | 1 (12.5%) | 0 (0.0%) |
|  |  | Oxfendazole Cohort 2 | 8 | 0 (0.0) | 0 (0.0%) | 0 (0.0%) | 1 (12.5%) | 0 (0.0%) |
|  |  | Oxfendazole Cohort 3 | 8 | 0 (0.0) | 0 (0.0%) | 0 (0.0%) | 3 (37.5%) | 0 (0.0%) |
|  |  | Pooled placebo | 6 | 0 (0.0) | 0 (0.0%) | 0 (0.0%) | 2 (33.3%) | 0 (0.0%) |
|  | D5 | Oxfendazole Cohort 1 | - | - | - | - | - | - |
|  |  | Oxfendazole Cohort 2 | - | - | - | - | - | - |
|  |  | Oxfendazole Cohort 3 | 8 | 0 (0.0) | 0 (0.0%) | 0 (0.0%) | 4 (50.0%) | 0 (0.0%) |
|  |  | Pooled placebo | 2 | 0 (0.0) | 0 (0.0%) | 0 (0.0%) | 1 (50.0%) | 0 (0.0%) |
|  | D8 | Oxfendazole Cohort 1 | - | - | - | - | - | - |
|  |  | Oxfendazole Cohort 2 | - | - | - | - | - | - |
|  |  | Oxfendazole Cohort 3 | 8 | 0 (0.0) | 0 (0.0%) | 0 (0.0%) | 3 (37.5%) | 0 (0.0%) |
|  |  | Pooled placebo | 2 | 0 (0.0) | 0 (0.0%) | 0 (0.0%) | 1 (50.0%) | 0 (0.0%) |
|  | D14 | Oxfendazole Cohort 1 | 8 | 0 (0.0) | 0 (0.0%) | 0 (0.0%) | 4 (50.0%) | 0 (0.0%) |
|  |  | Oxfendazole Cohort 2 | 8 | 0 (0.0) | 0 (0.0%) | 0 (0.0%) | 3 (37.5%) | 0 (0.0%) |
|  |  | Oxfendazole Cohort 3 | 8 | 0 (0.0) | 0 (0.0%) | 0 (0.0%) | 4 (50.0%) | 0 (0.0%) |
|  |  | Pooled placebo | 6 | 0 (0.0) | 0 (0.0%) | 0 (0.0%) | 3 (50.0%) | 0 (0.0%) |

Based on safety set.

Note: Participants who have been withdrawn from study or not attended a visit are not counted in the expected number. Only two placebo participants from Cohort 3 are expected at Day 3 – Day 8.

Please note that listings below only include abnormal coagulation results recorded after baseline.
